# White Matter Diffusion Properties in Chronic Temporomandibular Disorders: An Exploratory Analysis

**DOI:** 10.3389/fpain.2022.880831

**Published:** 2022-06-21

**Authors:** Alexandra S. Budd, Thi K. T. Huynh, Peter Seres, Christian Beaulieu, Susan Armijo-Olivo, Jacqueline Cummine

**Affiliations:** ^1^Neuroscience and Mental Health Institute, Faculty of Medicine and Dentistry, University of Alberta, Edmonton, AB, Canada; ^2^Faculty of Science, University of Alberta, Edmonton, AB, Canada; ^3^Department of Biomedical Engineering, University of Alberta, Edmonton, AB, Canada; ^4^Department of Physical Therapy, Faculty of Rehabilitation Medicine, University of Alberta, Edmonton, AB, Canada; ^5^Faculty of Business and Social Sciences, University of Applied Sciences Osnabrück, Osnabrück, Germany; ^6^Department of Dentistry, Faculty of Medicine and Dentistry, University of Alberta, Edmonton, AB, Canada; ^7^Department of Communication Sciences and Disorders, Faculty of Rehabilitation Medicine, University of Alberta, Edmonton, AB, Canada

**Keywords:** diffusion tensor imaging, temporomandibular disorders, chronic pain, white matter, quality of life, orofacial pain

## Abstract

**Objective:**

To determine differences in diffusion metrics in key white matter (WM) tracts between women with chronic temporomandibular disorders (TMDs) and age- and sex-matched healthy controls.

**Design:**

Cross sectional study compared diffusion metrics between groups and explored their associations with clinical variables in subjects with TMDs.

**Methods:**

In a total of 33 subjects with TMDs and 33 healthy controls, we performed tractography to obtain diffusion metrics (fractional anisotropy [FA], mean diffusivity [MD], radial diffusivity [RD], and axial diffusivity [AD]) from the cingulum near the cingulate gyrus (CGC), the cingulum near the hippocampus (CGH), the fornix, the anterior limb of the internal capsule (ALIC), the posterior limb of the internal capsule (PLIC), and the uncinate fasciculus (UF). We compared diffusion metrics across groups and explored the relationships between diffusion metrics and clinical measures (pain chronicity and intensity, central sensitization, somatization, depression, orofacial behavior severity, jaw function limitations, disability, and interference due to pain) in subjects with TMDs.

**Results:**

We observed differences in diffusion metrics between groups, primarily in the right side of the brain, with the right CGC having lower FA and the right UF having lower FA and higher MD and RD in subjects with TMDs compared to healthy controls. No clinical measures were consistently associated with diffusion metrics in subjects with TMDs.

**Conclusion:**

The UF showed potential microstructural damage in subjects with TMDs, but further studies are needed to confirm any associations between diffusion changes and clinical measures.

## Introduction

Temporomandibular disorders (TMDs) are musculoskeletal pain disorders that affect the masticatory muscles, the temporomandibular joint (TMJ), and related structures in the head and neck (1). TMDs occur twice as often in women than men (2), and patients often experience additional allodynia and hyperalgesia throughout the body (3). As the primary source of chronic orofacial pain (4), TMDs can cause pain for longer than 3 months and impact daily function, including cognitive (e.g., memory), motor, affective, social, work and overall quality of life (4–13).

Not surprisingly, patients with chronic TMDs have demonstrated higher rates of depression and somatization compared to patients with acute TMDs (14). Additionally, patients who do not respond to treatment have higher levels of catastrophizing than those who do respond to treatment, which underscores the relationship between pain-related activity interference and depression (15, 16). The variety of symptoms accompanying TMDs demonstrates a widespread effect on the central nervous system that can profoundly impact patients' well-being and points to the necessity of improving our understanding of these conditions (11, 17–32). The purpose of the current work was to examine the microstructural properties of white matter (WM) pathways in individuals with chronic TMDs and the explore the extent to which these measures of the central nervous system were related to pain chronicity and intensity measures (such as central sensitization, somatization, depression, orofacial behavior severity, jaw functional limitations, disability, and interference due to pain).

One helpful tool to study the effects of chronic pain on the central nervous system is Diffusion Tensor Imaging (DTI), which provides information about brain microstructure via the movement of water molecules within the tissue (33, 34). DTI provides four standard measures of diffusion (i.e., fractional anisotropy [FA], mean diffusivity [MD], axial diffusivity [AD], and radial diffusivity [RD]), and the inclusion of all measures for complex populations such as chronic pain are necessary to describe how chronic pain conditions affect brain structure (34). For instance, when comparing patients with a range of chronic musculoskeletal pain conditions to healthy controls, one study (35) found lower FA and AD in the cingulum adjacent to the hippocampus (CGH) and higher RD and AD in the internal capsule in patients with chronic pain. Moreover, FA and AD within the uncinate fasciculus (UF) were associated with pain experience, severity, and catastrophizing. Several studies (36–44) have also examined diffusivity in patients with trigeminal neuralgia, which causes orofacial pain; however, trigeminal neuralgia may have different neural consequences than TMDs since it is neuropathic (27), and thus, the extent to which such findings 1) are present across all four diffusion metrics and 2) generalize to patients with TMDs remains unknown.

In addition to comprehensive DTI metrics, there is a need for more studies on TMDs specifically. To our knowledge, there are only three studies that include analyses of diffusion changes within sensory, affective, and cognitive circuitry in patients with TMDs. One study (29) found that patients with TMDs had decreased FA and increased MD and RD compared to healthy controls in regions including the internal and external capsules, primary somatosensory and motor cortices, thalamus, and corpus callosum. Additionally, FA in the right internal capsule was negatively associated with pain intensity and unpleasantness. In another study (27), FA within the somatosensory cortex was comparable between patients with TMDs and healthy controls. Finally, another study (30) found that, for patients with TMDs, helplessness was positively correlated with FA in the cingulum and tracts connecting the somatosensory and premotor areas. In contrast, helplessness was negatively correlated with FA within motor-related regions such as the posterior limb of the internal capsule (PLIC). Overall, the novel and somewhat mixed findings with respect to FA and clinical outcomes in individuals with TMDs underscore the necessity for further investigation in this population.

This study aimed to investigate the differences in DTI metrics between patients with TMDs and healthy controls in tracts involved in the affective and cognitive aspects of chronic pain, including the cingulum near the cingulate gyrus (CGC), CGH, fornix, anterior limb of the internal capsule (ALIC), PLIC, and the UF. We hypothesized that subjects with TMDs would show microstructural alterations in these tracts compared to healthy controls. Furthermore, we predicted that these changes would be associated with pain chronicity and intensity measures (such as central sensitization, somatization, depression, orofacial behavior severity, jaw functional limitations, disability, and interference due to pain), which are known to impact the lives of those suffering from chronic pain.

## Materials and Methods

### Participants

A total of 33 female patients with TMDs (mean age = 32; SD = 11; range = 20 to 58) were recruited from the orofacial pain clinic at the University of Alberta (UofA) and from the local community at the UofA for an ongoing study on the effects of exercise therapy on TMD symptoms.

An experienced assessor interviewed participants and performed a physical assessment to determine eligibility. Individuals were included in the study if they were women aged between 18 and 60 years old at the time of MRI and were diagnosed with pain-related TMDs with muscle pain as a chief complaint (including both myogenous and mixed TMDs). TMDs were classified according to the new *Diagnostic Criteria for Temporomandibular Disorders* (DC/TMD) (45). In addition, subjects with TMDs must have experienced pain in the masticatory muscle for at least 3 months that was not affected by trauma, previous infection, or an active inflammatory cause within the last month. Participants also needed a moderate to severe baseline pain score of at least 30 mm on a 100 mm visual analog scale (VAS) (46). Exclusion criteria included pregnancy, diagnosis with any severe disease (including metabolic, rheumatoid, or vascular diseases), or any other chronic pain disorders (e.g., irritable bowel syndrome and fibromyalgia), substance abuse, abnormal neurological examination, or contraindication to MRI (e.g., metallic surgical implants). Individuals were also excluded if they were currently receiving treatment for TMDs, had received exercise therapy within 6 months before study entry, or had ever received electrotherapy (e.g., transcutaneous electrical nerve stimulation [TENS] or interferential current). Eligible participants were then informed of the nature of the research before being asked to sign an informed consent per the University of Alberta's policies on research using human subjects. The UofA ethics committee has approved this project.

### Control Subjects

We pooled scans for 33 healthy female control participants (mean age = 32; SD = 10; range = 20 to 61 years) from two studies. Nine of these controls were recruited from a similar study protocol as participants with TMDs (cohort A), while the data for the remaining 24 of the controls were obtained from a healthy aging study (47) (cohort B). The inclusion criteria from these studies were as follows: Cohort A Controls—All participants spoke English as their native or primary language, had either normal or corrected-to-normal vision, no contraindications to MRI testing, and age-appropriate scores on a non-verbal intelligence testing. Participants were excluded if they had a history of hearing impairment, stroke or neurological disorders (e.g., ADHD). Cohort B Controls—Participants had no neurological, psychiatric or developmental disabilities, significant head injuries or contraindications to MRI (neurological/psychiatric conditions include conditions diagnosed by a physician or if the participant is currently taking medication for this condition). They were recruited through advertising and provided written informed consent before study participation. Both studies were approved by the University of Alberta Human Research Ethics Board. Both studies used nearly identical protocols, with the only difference in the number of slices acquired, resulting in minor differences in repetition time (TR).

### Demographic and Clinical Variables

At baseline, we collected age and clinical variables from subjects with TMDs. Clinical variables (48) and questionnaires were collected using our web-protected platform (REDCAP). Patient age, weight, and height were recorded at the first MRI visit. At the first clinical visit, patients reported pain intensity using the Visual Analog Scale (VAS)-measured on a 0 mm to 100 mm scale (46) taken as an average of past-week and current pain. Additionally, patients were assessed to determine the specific TMD diagnosis and which side was the most affected (bilateral, right-predominant, or left-predominant). We also asked patients to report their medication [including selective serotonin reuptake inhibitors (SSRIs)] and whether they had other complaints, including neck pain, headache, shoulder pain, back pain, and whiplash.

Additionally, we used clinical scales to measure emotional functioning and the level of disability experienced due to TMDs. Scales included the Oral Behavior Checklist (TOBCL) (49), the Neck Disability Index (NDI) score (50), Limitations of Daily Functions (LODF) (51), the Jaw Functional Limitations Scale-20 (JFS-20) score to determine jaw function (52), the Central Sensitization Inventory (CSI) total score (53), the Patient Health Questionnaire-9 (PHQ-9) for depression (54), and the Patient Health Questionnaire-15 (PHQ-15) for somatization (45), and, finally, days in pain (within the last 6 months), days of disability interference (within the previous 30 days), characteristic pain intensity, interference score, and total disability points from the graded chronic pain scale (GCPS) (55).

To measure tenderness (pressure pain threshold [PPT]) in the masseter and temporalis muscles, physiotherapists used an algometer to apply increasing pressure to the skin above the muscle until the patient felt the first sensation of pain and signaled the therapist to remove the algometer by saying “yes” (56). PPT values are defined as the minimum applied pressure in kg/cm^2^ that causes pain. Furthermore, based on these PPT scores, the most sensitive side of each muscle was recorded as the side that had the lowest PPT measure. We also recorded patients' self-reported side of predominant jaw pain.

The tools employed in this study have been validated and shown to be reliable and responsive methods for evaluating patients with TMDs (45). They are part of the Research Diagnostic Criteria for TMD (DC/TMD), which is the gold standard for diagnosing and characterizing patients with TMDs. In the present study, we will refer to age, weight, and height as demographic variables and the remaining measures as clinical variables. Detailed descriptions of the tools used in this study are available upon request from the authors.

### Image Acquisition

MRI images were acquired at Peter S. Allen MRI Research Center at the University of Alberta on a 3T Siemens Prisma (Erlangen, Germany) using a 64-channel head coil. DTI images were acquired using multi-band spin-echo EPI sequence, with FoV 220 mm × 220 mm, matrix 148 × 148 (native resolution 1.5 × 1.5 mm^2^ interpolated to 0.75 × 0.75 mm^2^ in-plane on the scanner), TE = 64 ms, TR = 4,910 ms / 4,700 ms (cohort A/B), 94 or 90 slices (cohort A/B) with 1.5 mm thickness, acquired axial-oblique, parallel to AC-PC, GRAPPA factor 2, multi-band factor 2, with 6 b0 volumes and 30 directions of diffusion volumes at b = 1,000 s/mm^2^ and b = 2,000 s/mm^2^, in 6 min 15 s/5 min 59 s (cohort A/B). Only volumes with b = 0 s/mm^2^ and b = 1,000 s/mm^2^ were used for analysis. Anatomical images were acquired using T1-weighted 3D MPRAGE sequence at 1 × 1 × 1 mm^3^ resolution (TE = 2.21 ms, TR = 1,700 ms, TI = 880 ms, flip angle = 10 deg, GRAPPA factor 2, sagittal oblique), in 3 min 37 s (cohort A) or 0.87 × 0.87 × 0.85 mm^3^ resolution (TE = 2.37 ms, TR = 1,800 ms, TI = 900 ms, flip angle = 8 deg, GRAPPA factor 3, sagittal oblique) in 3 min 39 s (cohort B).

### Image Analysis

We first processed DWI images using NLSAM (https://github.com/samuelstjean/nlsam/) for stabilization (57) and de-noising (58) using all available volumes. Afterwards, only five b0 and all b1000 volumes were extracted for further analysis using ExploreDTI Version 4.8.6 (59), a graphical toolbox used to process, analyze, and visualize exploratory diffusion MRI data. Images were inspected visually for quality, looking for, but not limited to: gross subject motion, signal artifacts, signal dropouts, or missing data files. All images were of good quality and retained in the study. Preprocessing steps included signal drift correction using quadratic model, Gibbs ringing correction (5 b0; Lambda = 100; iteration = 100; step size = 0.01), registration between diffusion images and structural images, masking to remove non-diffusion weighted signal (kernel = 9; 0.5 for non-DWIs; 0.8 for DWIs), non-rigid EPI correction for distortions, and corrections for individual subject motion.

**Figure 1:**
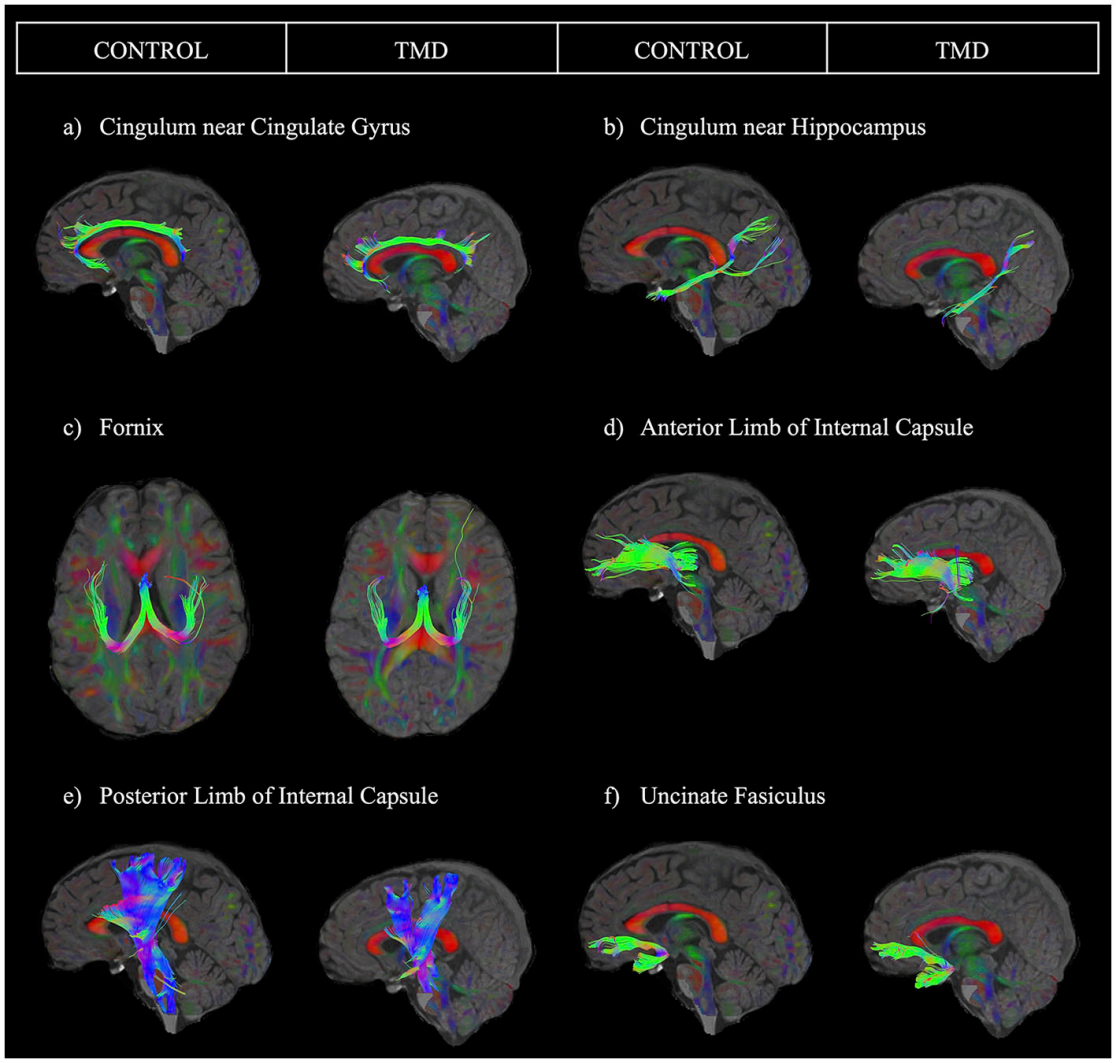
Sample images of each tract for one control participant and one participant with TMD. The sample tracts have been obtained from the left hemisphere. Tracts are presented as follows: **(a)** cingulum near the cingulate gyrus, **(b)** cingulum near the hippocampus, **(c)** fornix, **(d)** anterior limb of the internal capsule, **(e)** posterior limb of the internal capsule, and **(f)** uncinate fasciculus.

After processing the images, we performed region of interest (ROI) analyses, which generated specific tracts to extract the four diffusion parameters: FA, MD, AD, and RD. ROIs for tracking were drawn based on a template following the guidelines provided in a previous study and warped into the space of each brain for tracking in participant space (60). We used a minimum FA threshold of 0.20 and a maximum turning angle of 30 degrees to initiate and continue tracking for fiber tracking. We isolated the following tracts: cingulum near the cingulate gyrus (CGC), cingulum adjacent to the hippocampus (CGH), fornix, anterior limb of the internal capsule (ALIC), posterior limb of the internal capsule (PLIC), and uncinate fasciculus (UF) (see representative tracts in Figure 1). This tractography procedure was conducted by two trained assessors using standardized protocols as described below. A reliability analysis was conducted to determine inter- and intra-rater reliability for the two assessors. We describe this analysis in detail in the statistical analysis section below.

Tractography for the CGC and CGH was done following a previously described method (60), with a slight change. Since our images often did not capture the brainstem, we placed an “and” ROI next to the brainstem only when possible while performing tractography for the CGH. Tractography for the fornix was also done based on another protocol (61). For tractography of the ALIC, we placed three ROIs bilaterally: One “and” ROI around the internal capsule region, carefully avoiding the superior fronto-occipital fasciculus, one “not” ROI at the anterior of the splenium of the corpus callosum, and one “not” ROI approximately 13 slices inferior to the base of the “and” ROI. Additionally, we placed “not” ROIs as needed to remove any unrelated fibers. For the PLIC, we placed one “and” ROI and two “not” ROIs bilaterally. We placed the first “and” ROI at the first slice in which both the PLIC and the external capsule were clearly defined when moving the axial plane inferiorly starting at the top of the corpus callosum. We then placed two “not” ROIs: one ~10 slices anterior to the front of the “and” ROI, and the other just posterior to the splenium of the corpus callosum. Again, we placed additional “not” ROIs to remove extraneous fibers. Finally, we performed tractography of the UF following a protocol previously implemented by our lab (62).

After tractography was complete, we extracted FA, MD, AD (λ1), and RD ([λ2 + λ3]/2) values (unitless for FA and in mm^2^/s for MD, AD, and RD) from each tract for statistical analysis.

### Statistical Analysis

#### Demographic Comparisons

We compared demographic variables (i.e., age, weight, and height) between groups using 2-tailed independent samples *t*-tests.

#### Intra-Rater and Inter-rater Reliability Analyses

Each assessor performed tractography on half of the dataset (half of the controls and half of the patients). Therefore, to ensure tractography was comparable within and between assessors, each assessor repeated tractography on 10 anonymized participants from their initially assigned half of the dataset (intra-rater reliability) and then performed tractography on 10 anonymous participants from the other assessor's half of the dataset (inter-rater reliability). We then obtained intraclass correlation coefficients (ICCs) using two-way mixed-effects models with the absolute agreement and mean ratings (k = 2 raters; 2 occasions) for all analyses. Figure 1 depicts each tract analyzed for one control participant and one participant with TMD. Appendix Table 1 summarizes the ICC results. Intra-rater ICC values ranged from good (0.75–0.9) to excellent (above 0.9) for both raters for the left CGC, the right and left CGH, fornix, left ALIC, left and right PLIC, and left and right UF (63). For the right CGC, intra-rater values ranged from moderate (0.5–0.75) to excellent, and for the right ALIC, intra-rater values ranged from moderate to good. ICC values for inter-rater reliability were good to excellent for all tracts except the right ALIC, which had moderate reliability.

#### Models of DTI Metrics

The DTI measures are hierarchical, meaning that tracts (e.g., the CGC, CGH, fornix, ALIC, PLIC, and UF) are nested within each participant (i.e., the random effect). To account for the relationship within different levels of this hierarchy, we constructed multilevel models. This statistical technique has been suggested as the best strategy to account for correlated and hierarchical data (64). Because the fornix is unique as it is not separated into distinct left and right sides, we had to model the fornix separately. Additionally, while we obtained both demographic and clinical measures for the TMD group, we only had demographic variables available for the control group. Therefore, we constructed separate models using only the TMD group to determine the association between clinical variables and DTI metrics from each tract. In summary, we constructed four different mixed-level models to predict each DTI metric, considering: (1) all tracts excluding the fornix for both groups (TMD and controls), (2) only the fornix in both groups, (3) all tracts except for the fornix, only in the TMD group, and (4) only the fornix in only the TMD group. See Supplemental Material 1 for a more detailed description of these analyses.

We performed multilevel modeling using Stata 17.0 (65) and all other analyses using SPSS version 28.0 (66).

## Results

### Participant Demographics

Participant demographics and clinical characteristics are shown in Table 1. Overall, we observed no significant differences in demographics between participants with TMDs and control participants.

**Table 1:** Descriptive statistics of all participants of the study.

		**Control (*N* = 33)**	**TMD (*N* = 33)**	***t***	***p*** **-values**
Age (years), mean (SD)		31.85 (10.30)	32.21 (10.51)	−0.139	0.89
Weight (pounds), mean (SD)		154.00 (36.31)	153.82 (34.78)	0.021	0.983
Height (meters), mean (SD)		1.66 (0.07)	1.65 (0.06)	1.135	0.261
VAS (mm), mean (SD)	Current	–	43.33 (20.89)	–	–
	Past Week	–	51.18 (21.00)	–	–
	Average	–	47.26 (19.78)	–	–
TMJ Disorder—Left Side, frequency (percent)	None	–	21 (63.64)	–	–
	Disc Displacement with Reduction	–	12 (36.36)	–	–
	Disc Displacement with Reduction, with Intermittent Locking	–	0 (0.00)	–	–
TMJ Disorder—Right Side, frequency (percent)	None	–	24 (72.73)	–	–
	Disc Displacement with Reduction	–	8 (24.24)	–	–
	Disc Displacement with Reduction, with Intermittent Locking	–	1 (3.03)	–	–
Chief Complaint, frequency (percent)	Muscular TMD	–	14 (42.4)	–	–
	Mixed TMD	–	19 (57.6)	–	–
Pain Disorders, frequency (percent)	Myalgia	–	33 (100.00)	–	–
	Myofascial Pain with Referral	–	1 (3.03)	–	–
	Arthralgia—Left Side	–	24 (72.73)	–	–
	Arthralgia—Right Side	–	18 (54.55)	–	–
	Headache Attributed to TMD	–	1 (3.03)	–	–
Self-Reported Jaw Pain Side, frequency (percent)	Bilateral Jaw Pain	–	28 (84.8)	–	–
	Left	–	2 (6.1)	–	–
	Right	–	3 (9.1)	–	–
On SSRIs/Antidepressants, frequency (percent)	Yes	–	11 (33.3)	–	–
Other complaints—Neck, frequency (percent)	Neck pain	–	12 (36.4)	–	–
Other complaints—Headache, frequency (percent)	Headache	–	6 (18.2)	–	–
Other complaints—Shoulder, frequency (percent)	Shoulder pain	–	2 (6.1)	–	–
Other complaints—Back, frequency (percent)	Back pain	–	1 (3)	–	–
Other complaints—Whiplash, frequency (percent)	Whiplash	–	9 (27.3)	–	–
TOBCL total score, frequency (percent)	Middle orofacial behavior	–	1 (3)	–	–
	High orofacial behavior	–	32 (97)	–	–
TOBCL total score, mean (SD)	–	33.58 (7.44)	–	–	
PHQ-9 total score, frequency (percent)	None-minimal	–	9 (27.3)	–	–
	Mild	–	10 (30.3)	–	–
	Moderate	–	11 (33.3)	–	–
	Moderate Severe	–	2 (6.1)	–	–
	Severe	–	1 (3)	–	–
	Severe Disability	–	1 (3)	–	–
PHQ-9 total score, mean (SD)	–	8.73 (5.23)	–	–	
NDI total score, frequency (percent)	No Disability	–	1 (3)	–	–
	Mild Disability	–	17 (51.5)	–	–
	Moderate Disability	–	14 (42.4)	–	–
	Severe Disability	–	1 (3)	–	–
NDI total score, mean (SD)	–	14.27 (5.30)	–	–	
LODF total score, mean (SD)	–	14.55 (6.81)	–	–	
JFLS-20 total score, mean (SD)	–	45.39 (22.89)	–	–	
CSI total score, frequency (percent)	Subclinical CSP	–	2 (6.1)	–	–
	Mild CSL	–	3 (9.1)	–	–
	Moderate CSP	–	10 (30.3)	–	–
	Severe CSP	–	9 (27.3)	–	–
	Extreme CSP	–	4 (12.1)	–	–
CSI total score, mean (SD)	–	47.86 (9.92)			
PHQ-15 total score, frequency (percent)	Minimal Somatization	–	2 (6.1)	–	–
	Low Somatization	–	8 (24.2)	–	–
	Medium Somatization	–	17 (51.5)	–	–
	High Somatization	–	6 (18.2)	–	–
PHQ-15 total score, mean (SD)	–	11.33 (4.20)	–	–	
GCPS—Days in pain, mean (SD)	–	106.597 (65.49)	–	–	
GCPS—Disability interference days, mean (SD)	–	3.561 (6.23)	–	–	
GCPS—Pain intensity, mean (SD)	–	46.5657 (20.76)	–	–	
GCPS—Interference, mean (SD)	–	27.1717 (24.37)	–	–	
GCPS—Disability points, mean (SD)	–	1.67 (2.01)	–	–	
Most Sensitive Side—Temporalis Anterior, frequency (percent)	Bilateral	–	0 (0.0)	–	–
	Left	–	17 (51.5)	–	–
	Right	–	16 (48.5)	–	–
Most Sensitive Side—Temporalis Medial, frequency (percent)	Bilateral	–	1 (3.0)	–	–
	Left	–	8 (24.2)	–	–
	Right	–	24 (72.7)	–	–
Most Sensitive Side—Temporalis Posterior, frequency (percent)	Bilateral	–	1 (3.0)	–	–
	Left	–	20 (60.6)	–	–
	Right	–	12 (36.4)	–	–
Most Sensitive Side—Masseter Superior Anterior, frequency (percent)	Bilateral	–	2 (6.1)	–	–
	Left	–	12 (36.4)	–	–
	Right	–	19 (57.6)	–	–
Most Sensitive Side—Masseter Superior Inferior, frequency (percent)	Bilateral	–	1 (3.0)	–	–
	Left	–	17 (51.5)	–	–
	Right	–	15 (45.5)	–	–
Most Sensitive Side—Masseter Deep, frequency (percent)	Bilateral	–	1 (3.0)	–	–
	Left	–	18 (54.5)	–	–
	Right	–	14 (42.4)	–	–
PPT hand, mean (SD)		–	3.9 (1.22)	–	–
PPT temporalis anterior left, mean (SD)		–	1.77 (0.64)	–	–
PPT temporalis anterior right, mean (SD)		–	1.83 (0.72)	–	–
PPT temporalis medial left, mean (SD)		–	2.01 (0.77)	–	–
PPT temporalis medial right, mean (SD)		–	1.94 (0.78)	–	–
PPT temporalis posterior left, mean (SD)		–	2.12 (0.88)	–	–
PPT temporalis posterior right, mean (SD)		–	2.16 (0.81)	–	–
PPT masseter superior anterior left, mean (SD)		–	1.44 (0.59).	–	–
PPT masseter superior anterior right, mean (SD)		–	1.31 (0.49)	–	–
PPT masseter superior inferior left, mean (SD)		–	1.39 (0.62)	–	–
PPT masseter superior inferior right, mean (SD)		–	1.37 (0.52)	–	–
PPT masseter deep left, mean (SD)		–	1.53 (0.56)	–	–
PPT masseter deep right, mean (SD)		–	1.52 (0.59)	–	–

*VAS, Visual Analog Scale; TMJ, Temporomandibular Joint; TMD, Temporomandibular Disorder; TOBCL, Oral Behavior Checklist; PHQ-9, Patient Health Questionnaire-9; PHQ-15, Patient Health Questionnaire-15; NDI, Neck Disability Index; LODF, Limitations of Daily Functions; JFLS-20, Jaw Functional Limitations Scale-20; CSI, Central Sensitization Inventory; GCPS, Graded Chronic Pain Scale; PPT, Pressure Pain Threshold*.

### DTI Results

#### Models of DTI Metrics for Both Groups (TMD vs. Healthy) and All Tracts Except the Fornix

Appendix Table 2 shows the results of the univariate analyses and which demographic variables we used to build the models to predict DTI metrics for both groups in all tracts except the fornix.

**Figure 2:**
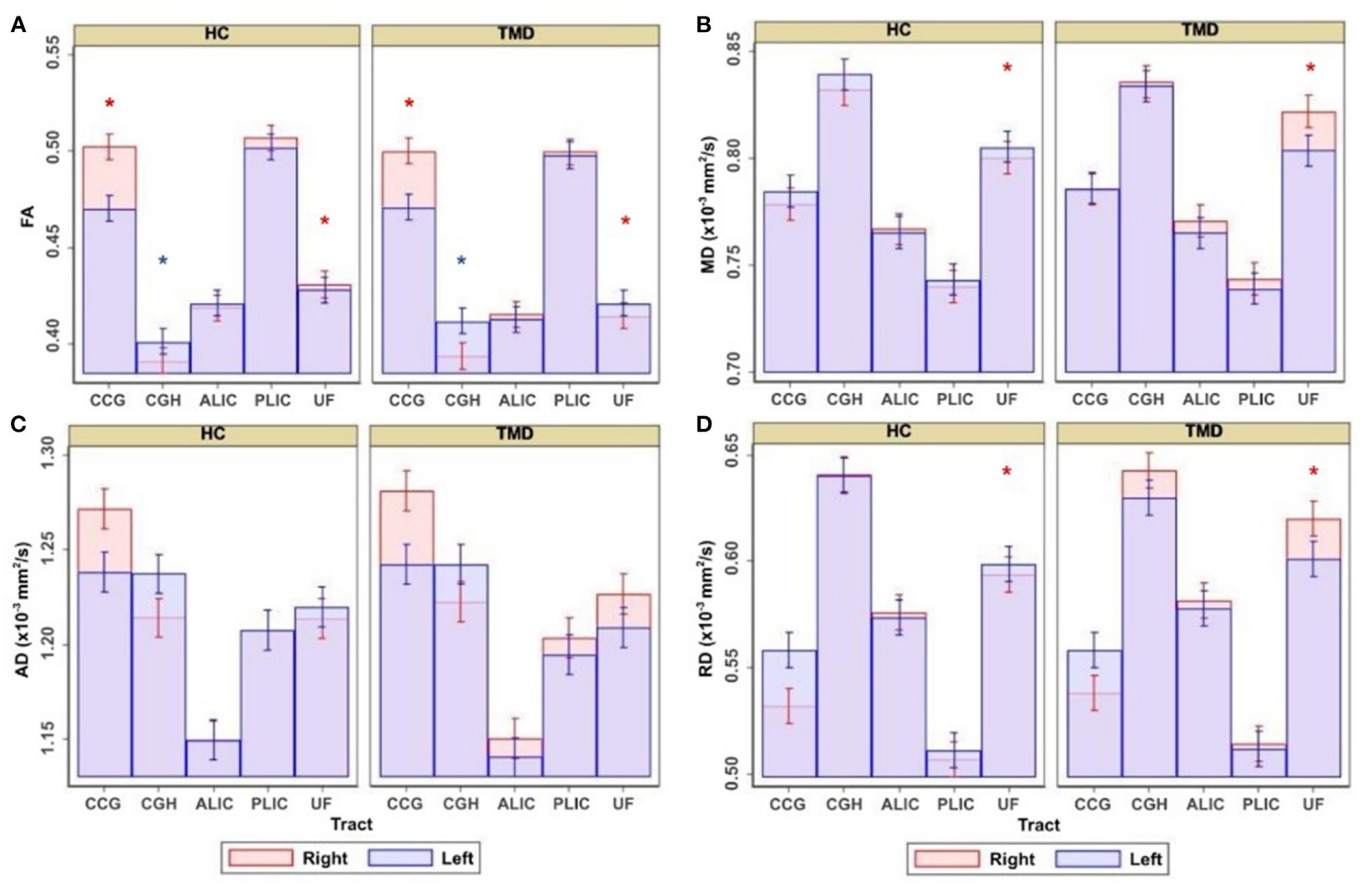
Adjusted estimates comparing healthy controls (HC) and subjects with temporomandibular disorder (TMD) for the cingulum near the cingulate gyrus (CGC), cingulum near the hippocampus (CGH), anterior limb of the internal capsule (ALIC), posterior limb of the internal capsule (PLIC), and uncinate fasciculus (UF). Estimates are presented for **(A)** FA, **(B)** MD, **(C)** AD, and **(D)** RD. Red asterisks denote significant differences between groups in the right side of the tract displayed underneath, and blue asterisks denote significant differences between groups in the left side of the tract.

Table 2 shows the coefficients, 95% CIs, and *p*-values for the demographic variables predicting the diffusion metrics, tracts relative to the CGC, subjects with TMDs relative to controls, and the left side relative to the right. No demographic variables were significantly associated with FA, but age was negatively associated with MD (*p* < 0.001), AD (*p* < 0.001), and RD (*p* < 0.001), while weight was positively associated with MD, AD, and RD (*p* = 0.002, *p* = 0.001, and *p* = 0.013, respectively). In this analysis, subjects with TMDs did not significantly differ from controls in any DTI metrics when adjusted by the demographic variables. However, a significant effect of “side” was found; the left side had significantly lower FA and AD and higher MD and RD than the right side. Additionally, the interaction between group and tract for the UF was significant (Figure 2).

**Table 2:** Fixed and random effects of the multilevel mixed-effects linear regression for the diffusion metrics (FA, MD, AD, and RD) for the models including both participant groups and all tracts except the fornix.

**Fixed effects**																
	**FA**				**MD**				**AD**				**RD**			
**Variable**	**Coef**.	**95% CI**		***p*** **-value**	**Coef. (×10^−3^)**	**95% CI (×10^−3^)**		***p*** **-value**	**Coef. (×10^**−3**^)**	**95% CI (×10^−3^)**		***p*** **-value**	**Coef. (×10^**−3**^)**	**95% CI (×10^−3^)**		***p*** **-value**
Age (years)	−0.0002	−0.0004;	0.0001	0.247	−0.0009	−0.0012;	−0.0005	<0.001^***^	−0.0015	−0.0020;	−0.0010	<0.001^***^	−0.0005	−0.0009;	−0.0001	0.009^**^
Weight (pounds)	NI	NI	NI	NI	0.0002	0.0001;	0.0003	0.002^**^	0.0002	0.0001;	0.0004	0.001^**^	0.0001	0.00003;	0.0003	0.013^*^
CGC	1				1				1				1			
CGH	0.0001	−0.1196;	−0.1027	<0.001^***^	0.0532	0.0460;	0.0605	0.002^**^	−0.0579	−0.0697;	−0.0462	<0.001^***^	0.1088	0.0998;	0.1179	<0.001^***^
ALIC	−0.1111	−0.0921;	−0.0752	<0.001^***^	−0.0117	−0.0190;	−0.0044	<0.001^***^	−0.1228	−0.1346;	−0.1110	<0.001^***^	0.0438	0.0348;	0.0529	<0.001^***^
PLIC	−0.0836	−0.0036;	0.0133	0.261	−0.0387	−0.0459;	−0.0314	<0.001^***^	−0.0647	−0.0765;	−0.0529	<0.001^***^	−0.0256	−0.0347;	−0.0166	<0.001^***^
UF	0.0048	−0.0798;	−0.0629	<0.001^***^	0.0216	0.0144;	0.0289	<0.001^***^	−0.0586	−0.0704;	−0.0468	<0.001^***^	0.0618	0.0527;	0.0708	<0.001^***^
Group (1 = HC, 2 = TMD)	−0.0713	−0.0117;	0.0073	0.657	0.0068	−0.0036;	0.0172	0.201	0.0090	−0.0059;	0.0239	0.237	0.0057	−0.0060;	0.0174	0.339
Side (1 = right, 2 = left)	−0.0022	−0.0384;	−0.0255	<0.001^***^	0.0061	−0.00003;	0.0123	0.051	−0.0335	−0.0453;	−0.0217	<0.001^***^	0.0260	0.0189;	0.0330	<0.001^***^
**Tract and Group interaction**																
*CGH + TMD Group*	0.0047	−0.0072;	0.0167	0.440	−0.0030	−0.0133;	0.0073	0.563	−0.0008	−0.0175;	0.0159	0.925	−0.0042	−0.0169;	0.0087	0.526
*ALIC + TMD Group*	−0.0012	−0.0131;	0.0108	0.845	−0.0030	−0.0132;	0.0073	0.575	−0.0077	−0.0244;	0.0085	0.363	−0.0005	−0.0133;	0.0123	0.933
*PLIC + TMD Group*	−0.0051	−0.0171;	0.0068	0.400	−0.0029	−0.0132;	0.0074	0.577	−0.0127	−0.0294;	0.0040	0.136	0.0020	−0.0108;	0.0148	0.764
*UF + TMD Group*	−0.0143	−0.0263;	−0.0024	0.019^*^	0.0149	0.0046;	0.0252	0.005^**^	0.0045	−0.0122;	0.0212	0.595	0.0201	0.0073;	0.0329	0.002^**^
**Random effects**																
	**FA**				**MD**				**AD**				**RD**			
	**Estimate**		**95% CI**		**Estimate (×10** ^**−3**^ **)**		**95% CI (×10** ^**−3**^ **)**		**Estimate (×10** ^**−3**^ **)**		**95% CI (×10** ^**−3**^ **)**		**Estimate (×10** ^**−3**^ **)**		**95% CI (×10** ^**−3**^ **)**	
Subject ID	0.00008		0.00005; 0.00014		0.0000002		0.0000002; 0.0000004		0.0000004		0.0000002; 0.0000005		0.0000002		0.0000002; 0.0000004	
*Variation in intercepts*																
*Variation in residuals*	–		–		–		–		0.0000006		0.0000005; 0.0000007		–		–	
Tract	0.00013		0.00009; 0.00017		0.0000001		0.00000004; 0.0000001		–		–		0.0000001		0.0000001; 0.0000002	
*Variation in intercepts*																
*Variation in residuals*	0.00018		0.00016; 0.00021		0.0000002		0.0000001; 0.0000002		–		–		0.0000002		0.0000002; 0.0000002	

*FA, Fractional Anisotropy; MD, Mean Diffusivity; AD, Axial Diffusivity; RD, Radial Diffusivity; CGC, Cingulum near Cingulate Gyrus; CGH, Cingulum near Hippocampus; ALIC, Anterior Limb of the Internal Capsule; PLIC, Posterior Limb of the Internal Capsule; UF, Uncinate Fasciculus*.

* *Indicates P < 0.05;*

** *Indicates P < 0.01;*

*** *Indicates P < 0.001*.

Although the groups were not different when diffusion metrics were pooled across several tracts, performing group comparisons allowed us to determine tract- and side-specific differences across groups. Table 3 and Figure 2A show that, compared to controls, subjects with TMDs have lower FA in the right CGC and right UF and higher FA in the left CGH. The TMD group also had higher MD in the right UF (Table 4; Figure 2B), no difference in AD (Table 5; Figure 2C), and higher RD (Table 6; Figure 2D) in the right UF.

**Table 3:** Differences comparing TMD vs. HC for FA (unitless) measured in all tracts excluding the fornix.

**Tract**	**Side**	**Group difference (Standard Error)**	**95% Confidence interval**
CGC	**Right**	**−0.032 (0.003)**	**−0.038; −0.025**
	Left	0.001 (0.005)	−0.009; 0.010
CGH	Right	0.003 (0.005)	−0.007; 0.012
	**Left**	**0.011 (0.005)**	**0.001; 0.020**
ALIC	Right	−0.003 (0.005)	−0.013; 0.006
	Left	−0.008 (0.005)	−0.018; 0.001
PLIC	Right	−0.007 (0.005)	−0.017; 0.002
	Left	−0.004 (0.005)	−0.014; 0.005
UF	**Right**	**−0.016 (0.005)**	**−0.026; −0.007**
	Left	−0.007 (0.005)	−0.016; 0.002

*TMD, Temporomandibular Disorder; HC, Healthy Control; FA, Fractional Anisotropy; CGC, Cingulum near Cingulate Gyrus; CGH, Cingulum near Hippocampus; ALIC, Anterior Limb of the Internal Capsule; PLIC, Posterior Limb of the Internal Capsule; UF, Uncinate Fasciculus. Significant differences as shown by the 95% confidence intervals are bolded*.

**Table 4:** Differences comparing TMD vs. HC for MD (mm^2^/s) measured in all tracts excluding the fornix.

**Tract**	**Side**	**Group difference (Standard Error) × 10^−3^ mm^2^/s**	**95% Confidence interval × 10^−3^ mm^2^/s**
CGC	Right	0.0068 (0.0031)	−0.00002; 0.0123
	Left	0.0013 (0.0053)	−0.0091; 0.0117
CGH	Right	0.0038 (0.0053)	−0.0067; 0.0142
	Left	−0.0054 (0.0053)	−0.0158; 0.0050
ALIC	Right	0.0039 (0.0053)	−0.0066; 0.0143
	Left	−0.0002 (0.0053)	−0.0106; 0.0102
PLIC	Right	0.0033 (0.0031)	−0.0028; 0.0095
	Left	0.0042 (0.0053)	−0.0146; 0.0063
UF	**Right**	**0.0217 (0.0053)**	**0.0113; 0.0321**
	Left	−0.0017 (0.0053)	−0.0121; 0.0088

*TMD, Temporomandibular Disorder; HC, Healthy Control; MD, Mean Diffusivity; CGC, Cingulum near Cingulate Gyrus; CGH, Cingulum near Hippocampus; ALIC, Anterior Limb of the Internal Capsule; PLIC, Posterior Limb of the Internal Capsule; UF, Uncinate Fasciculus. Significant differences as shown by the 95% confidence intervals are bolded*.

**Table 5:** Differences comparing TMD vs. HC for AD (mm^2^/s) measured in all tracts excluding the fornix.

**Tract**	**Side**	**Group difference (Standard Error) × 10^−3^ mm^2^/s**	**95% Confidence interval × 10^−3^ mm^2^/s**
CGC	Right	0.0090 (0.0076)	−0.0059; 0.0239
	Left	0.0039 (0.0076)	−0.0110; 0.0189
CGH	Right	0.0082 (0.0076)	−0.0067; 0.0231
	Left	0.0053 (0.0076)	−0.0096; 0.0202
ALIC	Right	0.0013 (0.0076)	−0.0137; 0.0162
	Left	−0.0093 (0.0076)	−0.0242; 0.0056
PLIC	Right	−0.0037 (0.0076)	−0.0186; 0.0112
	Left	−0.0128 (0.0076)	−0.0278; 0.0021
UF	Right	−0.0135 (0.0076)	−0.0014; 0.0284
	Left	−0.0106 (0.0076)	−0.0255; 0.0043

*TMD, Temporomandibular Disorder; HC, Healthy Control; AD, Axial Diffusivity; CGC, Cingulum near Cingulate Gyrus; CGH, Cingulum near Hippocampus; ALIC, Anterior Limb of the Internal Capsule; PLIC, Posterior Limb of the Internal Capsule; UF, Uncinate Fasciculus*.

**Table 6:** Differences comparing TMD vs. HC for RD (mm^2^/s) measured in all tracts excluding the fornix.

**Tract**	**Side**	**Group difference (Standard Error) × 10^−3^ mm^2^/s**	**95% Confidence interval × 10^−3^ mm^2^/s**
CGC	Right	0.0057 (0.0060)	−0.0060; 0.0174
	Left	−0.00002 (0.0060)	−0.0117; 0.0117
CGH	Right	0.0016 (0.0060)	−0.0101; 0.0132
	Left	−0.0107 (0.0060)	−0.0224; 0.0009
ALIC	Right	0.0052 (0.0060)	−0.0065; 0.0168
	Left	0.0043 (0.0060)	−0.0074; 0.0160
PLIC	Right	0.0077 (0.0060)	−0.0040; 0.0193
	Left	0.0002 (0.0060)	−0.0115; 0.0118
UF	**Right**	**0.0258 (0.0060)**	**0.0141; 0.0375**
	Left	0.0028 (0.0060)	−0.0089; 0.0145

*TMD, Temporomandibular Disorder; HC, Healthy Control; RD, Radial Diffusivity; CGC, Cingulum near Cingulate Gyrus; CGH, Cingulum near Hippocampus; ALIC, Anterior Limb of the Internal Capsule; PLIC, Posterior Limb of the Internal Capsule; UF, Uncinate Fasciculus. Significant differences as shown by the 95% confidence intervals are bolded*.

#### Models of DTI Metrics for Both Groups (TMD vs. Healthy): Fornix Only

Appendix Table 3 shows the results of the univariate analyses and which demographic variables we used to build the models to predict DTI for both groups in the fornix only.

Table 7 shows the coefficients, 95% CIs, and *p*-values for the demographic variables associated with the diffusion metrics and subjects with TMDs relative to controls. Age was significantly negatively associated with FA (*p* = 0.002) and positively associated with MD, AD, and RD (*p* = 0.008, *p* = 0.037, and *p* = 0.004, respectively). In this analysis, subjects with TMDs did not significantly differ from controls in any DTI metrics.

**Table 7:** Fixed and random effects of the multilevel mixed-effects linear regression for the diffusion metrics (FA, MD, AD, and RD) for models including both participant groups and only the fornix.

**Fixed effects**												
	**FA**			**MD**			**AD**			**RD**		
**Variable**	**Coef**.	**95% CI**	***p*** **-value**	**Coef. (×10^**−3**^)**	**95% CI (×10^**−3**^)**	***p*** **-value**	**Coef. (×10^**−3**^)**	**95% CI (×10^**−3**^)**	***p*** **-value**	**Coef. (×10^**−3**^)**	**95% CI (×10^**−3**^)**	***p*** **-value**
Age (years)	−0.0008	−0.0012; −0.0003	0.002^**^	0.0025	0.0007; 0.0044	0.008^**^	0.0024	0.0001; 0.0046	0.037^*^	0.0026	0.0009; 0.0044	0.004^**^
Group (1 = HC, 2 = TMD)	0.0001	−0.0097; 0.0099	0.991	0.0011	−0.0373; 0.0395	0.955	0.0022	−0.0438; 0.0482	0.926	0.0006	−0.0353; 0.0364	0.975
**Random effects**												
	**FA**			**MD**			**AD**			**RD**		
	**Estimate**	**95% CI**		**Estimate (×10** ^**−3**^ **)**	**95% CI (×10** ^**−3**^ **)**		**Estimate (×10** ^**−3**^ **)**	**95% CI (×10** ^**−3**^ **)**		**Estimate (×10** ^**−3**^ **)**	**95% CI (×10** ^**−3**^ **)**	
*Variation in residuals*	0.00041	0.00029; 0.00058		0.000006	0.000005; 0.000009		0.000009	0.000006; 0.000013		0.000006	0.000004; 0.000008	

*FA, Fractional Anisotropy; MD, Mean Diffusivity; AD, Axial Diffusivity; RD, Radial Diffusivity*.

* *Indicates P < 0.05;*

** *Indicates P < 0.01*.

#### Models of DTI Metrics for Subjects With TMDs Only and All Tracts Except the Fornix

Appendix Table 4 shows the univariate analyses' results and the clinical and demographic variables that we used to build the models to predict DTI metrics in only the TMD group for all tracts except the fornix.

Table 8 shows the coefficients, 95% CIs, and *p*-values for the demographic and clinical variables predicting, and associated with, the diffusion metrics and other tracts relative to the CGC and the left side relative to the right. For FA, PPT of the right medial temporalis was significantly positively associated with FA (*p* = 0.010). Age (*p* < 0.001) and headaches (*p* = 0.031) were significantly negatively associated with MD. AD was negatively associated with age (*p* < 0.001) and positively associated with weight (*p* = 0.005), while RD was negatively associated with age (*p* = 0.014).

**Table 8:** Fixed and random effects of the multilevel mixed-effects linear regression for the diffusion metrics (FA, MD, AD, and RD) for models with the TMD group only and all tracts except the fornix.

	**FA**			**MD**			**AD**			**RD**		
**Variable**	**Coef**.	**95% CI**	***p*** **-value**	**Coef. (×10^**−3**^)**	**95% CI (×10^**−3**^)**	***p*** **-value**	**Coef. (×10^**−3**^)**	**95% CI (×10^**−3**^)**	***p*** **-value**	**Coef. (×10^**−3**^)**	**95% CI (×10^**−3**^)**	***p*** **-value**
Age (years)	−0.0001	−0.0004; 0.0002	0.392	−0.0010	−0.0015; −0.0005	<0.001^***^	−0.0020	−0.0026; −0.0026	<0.001^***^	−0.0006	−0.0011; −0.0001	0.014^*^
Weight (pounds)	NI	NI	NI	NI	NI	NI	0.0003	0.0001; 0.0006	0.005^**^	NI	NI	NI
Other complaints — Headache (0 = no, 1 = yes)	NI	NI	NI	−0.0146	−0.0279; −0.0013	0.031^*^	NI	NI	NI	NI	NI	NI
Antidepressant (0 = no, 1 = yes)	NI	NI	NI	NI	NI	NI	0.0055	−0.0107; 0.0216	0.507	NI	NI	NI
PPT temporalis medial right	0.0050	0.0012; 0.0088	0.010^*^	NI	NI	NI	NI	NI	NI	NI	NI	NI
CGC	1			1			1			1		
CGH	−0.1064	−0.1151; −0.0977	<0.001^***^	0.0502	0.0432; 0.0572	<0.001^***^	−0.0588	−0.0710; −0.0465	<0.001^***^	0.1047	0.0957; 0.1136	<0.001^***^
ALIC	−0.0848	−0.0935; −0.0761	<0.001^***^	−0.0147	−0.0217; −0.0076	<0.001^***^	−0.1306	−0.1428; −0.1183	<0.001^***^	0.0433	0.0343; 0.0522	<0.001^***^
PLIC	−0.0003	−0.0090; 0.0084	0.950	−0.0416	−0.0486; −0.0346	<0.001^***^	−0.0774	−0.0897; −0.0652	<0.001^***^	−0.0237	−0.0326; −0.0147	<0.001^***^
UF	−0.0856	−0.0944; −0.0769	<0.001^***^	0.0366	0.0295; 0.0436	<0.001^***^	−0.0541	−0.0663; −0.0418	<0.001^***^	0.0819	0.0729; 0.0908	<0.001^***^
Side (1 = right, 2 = left)	−0.0291	−0.0355; −0.0227	<0.001^***^	0.0006	−0.0058; 0.0070	0.849	−0.0386	−0.0490; −0.0282	<0.001^***^	0.0202	0.0130; 0.0275	<0.001^***^
**Random effects**												
	**FA**			**MD**			**AD**			**RD**		
	**Estimate**	**95% CI**		**Estimate (×10** ^**−3**^ **)**	**95% CI (×10** ^**−3**^ **)**		**Estimate (×10** ^**−3**^ **)**	**95% CI (×10** ^**−3**^ **)**		**Estimate (×10** ^**−3**^ **)**	**95% CI (×10** ^**−3**^ **)**	
Subject ID												
*Variation in intercepts*	0.00002	0.00001; 0.00011		0.0000002	0.0000001; 0.0000003		0.0000003	0.0000002; 0.0000005		0.0000002	0.0000001; 0.0000003	
*Variation in residuals*	–	–		–	–		–	–		–	–	
Tract	0.00015	0.00010; 0.00023		0.00000004	0.00000002; 0.00000010		0.0000002	0.0000001; 0.0000003		0.0000001	0.0000001; 0.0000002	
*Variation in intercepts*												
*Variation in residuals*	0.00018	0.00014; 0.00022		0.00000017	0.00000014; 0.00000022		0.0000005	0.0000004; 0.0000006		0.0000002	0.0000002; 0.0000003	

*FA, Fractional Anisotropy; MD, Mean Diffusivity; AD, Axial Diffusivity; RD, Radial Diffusivity; CGC, Cingulum near Cingulate Gyrus; CGH, Cingulum near Hippocampus; ALIC, Anterior Limb of the Internal Capsule; PLIC, Posterior Limb of the Internal Capsule; UF, Uncinate Fasciculus; PPT, Pain Pressure Threshold; NI, Not included*.

* *Indicates P < 0.05;*

** *Indicates P < 0.01;*

*** *Indicates P < 0.001*.

#### Models of DTI Metrics for Subjects With TMDs: Fornix Only

Appendix Table 5 shows the results of the univariate analyses and which clinical and demographic variables we used to predict DTI metrics in only the TMD group for the fornix only.

Table 9 shows the coefficients, 95% CIs, and *p*-values for the demographic and clinical variables included in the models predicting the diffusion metrics in subjects with TMDs. Age, back pain, and SSRI use were all negatively associated with FA (*p* = 0.030, *p* = 0.029, and *p* = 0.011, respectively). MD was positively associated with SSRI use and PPT of the right superior-inferior masseter (*p* < 0.001 and *p* = 0.024, respectively), and AD was positively associated with SSRI use and PPT of the right superior-inferior masseter (*p* < 0.001 and *p* = 0.024, respectively). Finally, RD was positively associated with SSRI use and PPT of the right superior anterior masseter (*p* < 0.001 and *p* = 0.032, respectively).

**Table 9:** Fixed and random effects of the multilevel mixed-effects linear regression for the diffusion metrics (FA, MD, AD, and RD) for models including the TMD group only and only the fornix.

	**FA**			**MD**			**AD**			**RD**		
**Variable**	**Coef**.	**95% CI**	***p*** **-value**	**Coef. (×10^**−3**^)**	**95% CI (×10^**−3**^)**	***p*** **-value**	**Coef. (×10^**−3**^)**	**95% CI (×10^**−3**^)**	***p*** **-value**	**Coef. (×10^**−3**^)**	**95% CI (×10^**−3**^)**	***p*** **-value**
Age (years)	−0.0005	−0.0009; −0.00005	0.030^*^	0.0013	−0.0004; 0.0031	0.141	0.0011	−0.0012; 0.0034	0.355	0.0015	−0.0001; 0.0031	0.068
Other complaints—Back (0 = no, 1 = yes)	−0.0305	−0.0578; −0.0032	0.029^*^	NI	NI	NI	NI	NI	NI	NI	NI	NI
Antidepressant (0 = no, 1 = yes)	−0.0130	−0.0229; −0.0030	0.011^*^	0.0877	0.0489; 0.1265	<0.001^***^	0.1027	0.0529; 0.1526	<0.001^***^	0.0798	0.0448; 0.1148	<0.001^***^
PPT masseter superior anterior right	NI	NI	NI	NI	NI	NI	NI	NI	NI	0.0375	0.0033; 0.0717	0.032^*^
PPT masseter superior inferior right	NI	NI	NI	0.0410	0.0054; 0.0767	0.024^*^	0.0528	0.0071; 0.0985	0.024^*^	NI	NI	NI
**Random effects**												
	**FA**			**MD**			**AD**			**RD**		
	**Estimate**	**95% CI**		**Estimate (×10** ^**−3**^ **)**	**95% CI (×10** ^**−3**^ **)**		**Estimate (×10** ^**−3**^ **)**	**95% CI (×10** ^**−3**^ **)**		**Estimate (×10** ^**−3**^ **)**	**95% CI (×10** ^**−3**^ **)**	
*Variation in residuals*	0.00018	0.00011; 0.00029		0.000003	0.000002; 0.000005		0.000005	0.000003; 0.000008		0.000002	0.000001; 0.000004	

*FA, Fractional Anisotropy; MD, Mean Diffusivity; AD, Axial Diffusivity; RD, Radial Diffusivity; PPT, Pain Pressure Threshold; NI, Not included*.

* *Indicates P < 0.05;*

*** *Indicates P < 0.001*.

## Discussion

In this study, we investigated (1) differences in DTI metrics (FA, MD, AD, and RD) between patients with TMDs and healthy controls in WM tracts (the CGC, CGH, fornix, ALIC, PLIC, and UF) involved in the affective and cognitive aspects of chronic pain, and (2) relationships between DTI metrics and clinical variables in patients with TMDs. Two main findings emerged. First, when compared with controls, participants with TMDs had alterations in the cingulum (right-CGC and left-CGH) and right-UF. Second, clinical PPT values showed associations with diffusion metrics across all tracts for participants with TMDs. We discuss these findings, the potential implications, and areas of future study in the sections below.

### Differences Between Groups

FA in the right CGC was lower in subjects with TMDs than controls, while we observed the opposite relationship for the left CGH. The finding of lower FA in the right CGC is consistent with previous studies showing that the cingulate gyrus is involved in pain (67). Currently, there is a lack of literature associating WM characteristics of the CGC and CGH with TMDs, which is one area we sought to address with the present study. One study (35) of individuals with musculoskeletal pain did find a difference in FA for left CGH. However, FA for the left CGH in control subjects was higher than in participants with pain (35). Additionally, when measuring RD, they found several differences in right ALIC and right PLIC, which we did not observe (35). Another study (68) also found significantly lower FA in the cingulate gyrus and UF in patients with chronic migraines. These associations were mainly on the right side, similar to our findings. While our findings must be interpreted with caution as there were no converging findings (i.e., unlike the UF that showed several diffusion metrics were different between the groups) and there are few studies on TMDs available to which we can compare our results, it does appear that WM microstructure of the cingulum likely plays a role in chronic TMDs. Ultimately, more work is needed to better comprehend the role of the CGC and CGH in chronic musculoskeletal pain before we can make any definitive claims.

Notably, we found differences in multiple diffusivity measures between groups in the UF, with lower FA and higher MD and RD for participants with TMDs compared to controls. In general, decreased FA values may reflect a decrease in the linearity of water flow through axons and have often been associated with reduced axonal integrity (69). Additionally, MD reflects the rate of water diffusion in all axon directions, and higher MD values may relate to axonal damage and inflammation (69). While FA indicates disrupted diffusion along an axon and MD can provide a general indication of the magnitude of diffusion, AD and RD add information about the direction in which diffusion is disrupted (69). AD is a measure of diffusivity parallel to fiber tracts, whereas RD represents diffusivity perpendicular to fiber tracts. While demyelination may alter RD, it does not seem to affect AD (70), which may reflect axonal damage instead (71). Therefore, our results suggest that the right UF could have microstructural changes due to inflammation and potentially demyelination in individuals with TMDs, consistent with the finding that neuroinflammation is associated with depression and chronic pain (72). Accordingly, the UF may play an essential role in chronic pain conditions.

Another important finding in our study was the predominance of microstructural changes within the right hemisphere. These findings are intriguing because, in our cohort, most subjects with TMDs reported bilateral jaw pain, and the most sensitive side of the muscles as measured by PPT did not reveal any consistent lateralization of sensitivity. Other investigators have already observed a right-sided bias in the brain's response to pain. One study (73) that specifically investigated the lateralization of pain found strong evidence for increased pain-associated activity in the right hemisphere. This study proposes that this effect could be related to attention, which is a function strongly lateralized to the right hemisphere (74). Previous research (75) has also shown a predominantly right-hemisphere response when attending to a noxious stimulus. Therefore, it is plausible that sustained attention to pain caused by chronic pain could be at least partially responsible for the microstructural alterations seen predominantly in the right hemisphere of subjects with TMDs when compared to healthy controls.

### Associations With Clinical Variables

Models including only subjects with TMDs allowed us to determine the association between demographic and clinical variables and diffusion metrics in this group. PPT values showed associations with diffusion metrics across all tracts. The right medial temporalis PPT was positively associated with FA in the model of all tracts except the fornix, and PPT for the right superior anterior masseter was positively associated with MD, AD, and RD in the model fornix. These findings are conflicting because increased PPT values indicate higher pain thresholds (decreased sensitivity). In our study, PPT values were associated with increased WM integrity as measured by FA, but decreased WM integrity as measured by the other metrics. Overall, we did not find any tract consistently associated with PPT. To our knowledge, few studies have associated diffusion tensor imaging with PPT measures. However, one study (76) of subjects with chronic neck pain and whiplash-associated disorders also did not find any association between PPT and diffusion metrics (FA, MD, and RD) in the tracts examined (CGH and tapetum).

The UF was significantly different between groups in the present study, but no clinical measures were consistently associated with diffusivity in any tracts. However, a previous study (35) found an association between FA and AD in the UF and pain intensity, severity, and catastrophizing. This disparity in findings could be due to differences in the variables measured, heterogeneity of the samples (they included men and women with a range of musculoskeletal disorders), and sample size (they had a slightly larger sample). Although we found no evidence of a relationship between UF metrics and pain outcomes, our findings still suggest that there may be UF alterations associated with chronic pain states. Since we did not measure catastrophizing, we cannot determine whether this would contribute to differences in UF diffusivity; however, pain catastrophizing is known to be related to anxiety and depression (77). As seen in our sample, SSRI use (to treat anxiety and depression) was widely associated with microstructural alterations in the fornix and contributed to the model predicting AD in all tracts except the fornix, even though the association between AD and SSRI use did not reach significance. These findings hint at a potential association between diffusion metrics and anxiety and depression but are by no means conclusive.

### Strengths and Limitations

The present study has several strengths. One strength was the use of multilevel modeling, which allowed us to account for associations between the levels of organization of the tracts within the brains of subjects and provide more accurate estimates than would be achieved had we not accounted for the nested organization of our data (64). Additionally, we chose to explore AD and RD diffusion metrics, which are often missing in studies of musculoskeletal pain but can provide meaningful information about WM changes (34). Another strength was age- and sex-matched controls, which allowed us to identify differences between the groups while minimizing differences due to age and sex.

Although this study has many strengths, limitations must also be acknowledged. One limitation in this study is the lack of clinical variables collected from control participants. Because of this limitation, we could not determine if the associations between clinical variables and diffusion are unique to patients with TMDs or would also be found in healthy controls. However, since many of these variables are specific to chronic pain, anxiety, or depression, we expect these measures to be absent or minimal in healthy controls. Our study is also limited by the lack of homogeneity within our TMD group. While we restricted our selection criteria to include only individuals with mixed and myogenous TMDs, there may still be differences between these groups, even among individuals with varying degrees of referred pain. Our sample size did not allow us to explore sensitivity analyses by TMD diagnosis groups.

### Implications for Research and Practice

Future studies can expand on this research in various ways. For example, the longitudinal effects of TMDs are of interest. Future studies would benefit from studying the impact of chronic TMDs on the brain over an extended period. In addition, it is crucial to determine whether treatment strategies could potentially target and modify brain abnormalities seen in patients with TMDs. Our results support the growing evidence that chronic pain can alter the brain's structure and have profound emotional effects. Treatment for patients with chronic pain should focus on determining effective methods for minimizing the experience of pain in the affected areas and providing emotional support when needed.

## Conclusions

Our study provides evidence for alterations in microstructural integrity associated with chronic TMDs, especially in the UF. Furthermore, while we found evidence that sensitivity within the muscles as measured by PPT may be affected by TMDs, no PPT measures or other clinical measures were consistently associated with tract microstructure. Further studies are needed to determine how previously reported findings between pain and DTI metrics are reliable.

## Data Availability Statement

The raw data supporting the conclusions of this article will be made available by the authors, without undue reservation.

## Ethics Statement

The studies involving human participants were reviewed and approved by The University of Alberta Human Research Ethics Board. The patients/participants provided their written informed consent to participate in this study.

## Author Contributions

AB, SA-O, and JC provided substantial contributions to the conception and design of the work, participated in the analysis and interpretation of data, drafted the manuscript, and revised it critically for important intellectual content. AB and TH performed tractography and along with PS, CB, and JC participated in the acquisition, analysis, interpretation of data, drafted the manuscript, and revising it critically for important intellectual content. All authors read and approved the final version of the manuscript.

## Funding

This study was funded by an Innovation grant from The Women and Child Research Institute (WCHRI) from The University of Alberta, Canada, Fund for Dentistry, and the Dentistry Chair's Excellence Fund, School of Dentistry, University of Alberta.

## Conflict of Interest

The authors declare that the research was conducted in the absence of any commercial or financial relationships that could be construed as a potential conflict of interest.

## Publisher's Note

All claims expressed in this article are solely those of the authors and do not necessarily represent those of their affiliated organizations, or those of the publisher, the editors and the reviewers. Any product that may be evaluated in this article, or claim that may be made by its manufacturer, is not guaranteed or endorsed by the publisher.

## Abbreviations

AD: Axial Diffusivity
ALIC: Anterior Limb of Internal Capsule
CGC: Cingulum near the Cingulate Gyrus
CGH: Cingulum near the Cingulate Hippocampus
CNV: Cranial Nerve V (Trigeminal nerve)
CSI: Central Sensitization Inventory
DC/TMD: Diagnostic Criteria for Temporomandibular Disorders
DTI: Diffusion Tensor Imaging
FA: Fractional Anisotropy
GCPS: Graded Chronic Pain Scale
ICC: Intraclass Correlation Coefficient
JFLS-20: Jaw Functional Limitations Scale-20
LODF: Limitations of Daily Functions
MD: Mean Diffusivity
MRI: Magnetic Resonance Imaging
NDI: Neck Disability Index
PLIC: Posterior Limb of Internal Capsule
PHQ-9: Patient Health Questionnaire-9
PHQ-15: Patient Health Questionnaire-15
PPT: Pressure Pain Threshold
RD: Radial Diffusivity
ROI: Region of Interest
SSRI: Selective serotonin reuptake inhibitors
TENS: Transcutaneous electrical nerve stimulation or interferential current
TMD: Temporomandibular Disorders
TMJ: Temporomandibular Joint
TOBCL: Oral Behavior Checklist
UF: Uncinate Fasciculus
VAS: Visual Analog Scale
WM: White Matter.
