# Supplementary material for: White Matter Diffusion Properties in Chronic Temporomandibular Disorders: An Exploratory Analysis
Source: Front Pain Res (Lausanne). 2022 Jun 21;3:880831. doi: 10.3389/fpain.2022.880831 (PMC9254396; doi:10.3389/fpain.2022.880831)
Supplement: Supplementary file 1 [file Data_Sheet_1.pdf]

**Appendix Table 1: ICC Values**

| | **Intra-Reliability** | | | | | | | | **Inter-Reliability** | | | |
|---|---|---|---|---|---|---|---|---|---|---|---|---|
| | **Rater 1** | | | | **Rater 2** | | | | | | | |
| | **Average measures** | **95% CI Lower Bound** | **95% CI Upper Bound** | **F (df1 = 9, df2 = 9)** | **Average measures** | **95% CI Lower Bound** | **95% CI Upper Bound** | **F (df1 = 9, df2 = 9)** | **Average measures** | **95% CI Lower Bound** | **95% CI Upper Bound** | **F (df1 = 19, df2 = 19)** |
| CGC.l | 1.00 | 0.99 | 1.00 | 289.99 | 1.00 | 1.00 | 1.00 | 921.48 | 0.89 | 0.72 | 0.96 | 9.19 |
| CGC.r | 0.99 | 0.94 | 1.00 | 64.21 | 0.60 | -0.47 | 0.90 | 2.53 | 0.96 | 0.89 | 0.98 | 21.99 |
| CGH.l | 0.88 | 0.54 | 0.97 | 9.70 | 0.82 | 0.27 | 0.96 | 5.23 | 0.86 | 0.66 | 0.95 | 7.57 |
| CGH.r | 0.98 | 0.92 | 1.00 | 83.74 | 0.96 | 0.85 | 0.99 | 24.77 | 0.81 | 0.52 | 0.92 | 5.67 |
| Fornix | 0.98 | 0.90 | 0.99 | 37.37 | 0.96 | 0.84 | 0.99 | 34.00 | 0.95 | 0.87 | 0.98 | 19.68 |
| ALIC.l | 0.99 | 0.95 | 1.00 | 79.08 | 0.93 | 0.72 | 0.98 | 13.06 | 0.90 | 0.75 | 0.96 | 10.17 |
| ALIC.r | 0.70 | -0.27 | 0.93 | 3.19 | 0.85 | 0.36 | 0.96 | 6.01 | 0.75 | 0.38 | 0.90 | 4.10 |
| PLIC.l | 1.00 | 0.99 | 1.00 | 609.09 | 0.91 | 0.64 | 0.98 | 14.14 | 0.96 | 0.84 | 0.99 | 37.86 |
| PLIC.r | 1.00 | 0.99 | 1.00 | 584.33 | 0.96 | 0.84 | 0.99 | 22.84 | 0.99 | 0.98 | 1.00 | 140.39 |
| UF.l | 1.00 | 0.99 | 1.00 | 493.03 | 1.00 | 0.99 | 1.00 | 515.78 | 1.00 | 0.99 | 1.00 | 239.25 |
| UF.r | 1.00 | 1.00 | 1.00 | 1956.07 | 0.98 | 0.91 | 0.99 | 41.44 | 0.99 | 0.96 | 0.99 | 69.04 |

CGC: Cingulum near Cingulate Gyrus; CGH: Cingulum near Hippocampus; ALIC: Anterior Limb of the Internal Capsule; PLIC: Posterior Limb of the Internal Capsule; UF: Uncinate Fasciculus.

**Appendix Table 2**: Results of the univariate linear regression for variables that could influence diffusion metrics (FA, MD, AD, and RD) in all tracts excluding the fornix. Variables that were significant were candidates for use in the models including both groups and all tracts except the fornix.

| | **FA** | | | **MD** | | | **AD** | | | **RD** | | |
|---|---|---|---|---|---|---|---|---|---|---|---|---|
| **Variable** | **Coef. ($x10^{-4}$)** | **95% CI ($x10^{-4}$)** | **p-value** | **Coef. ($x10^{-7}$)** | **95% CI ($x10^{-7}$)** | **p-value** | **Coef. ($x10^{-7}$)** | **95% CI ($x10^{-)7}$)** | **p-value** | **Coef. ($x10^{-7}$)** | **95% CI ($x10^{-7}$)** | **p-value** |
| Age (years) | -1.58 | -5; 1.83 | 0.36 | -7.91 | -10.9; -4.96 | <0.001* | -14.10 | -17.8; -10.4 | <0.001* | -4.83 | -8.64; -1.02 | 0.01* |
| Weight (pounds) | -0.28 | -1.28; 0.72 | 0.59 | 1.53 | 0.66; 2.4 | <0.001* | 1.93 | 0.81; 3.05 | <0.001* | 1.33 | 0.21; 2.44 | 0.02* |
| Height (meters) | -146.17 | -695.14; 402.8 | 0.60 | 454.00 | -28.4; 937 | 0.07*† | 508.00 | -111; 1127 | 0.11*† | 427.00 | -187; 1041 | 0.17*† |

FA: Fractional Anisotropy; MD: Mean Diffusivity; AD: Axial Diffusivity; RD: Radial Diffusivity.
* Variable had a p-value of ≤ 0.20 and was a candidate for entry into the model; † Variable did not remain significant when entered in model.

**Appendix Table 3**: Results of the univariate linear regression for variables that could influence diffusion metrics (FA, MD, AD, and RD) in the fornix only. Variables that were significant were candidates for use in the models including both groups and only the fornix.

| | FA | | | MD | | | AD | | | RD | | |
|---|---|---|---|---|---|---|---|---|---|---|---|---|
| **Variable** | **Coef. (x10$^{-4}$)** | **95% CI (x10$^{-4}$)** | **p-value** | **Coef. (x10$^{-7}$)** | **95% CI (x10$^{-7}$)** | **p-value** | **Coef. (x10$^{-7}$)** | **95% CI (x10$^{-)7}$)** | **p-value** | **Coef. (x10$^{-7}$)** | **95% CI (x10$^{-7}$)** | **p-value** |
| Age (years) | -7.66 | -12.44; -2.88 | 0.002* | 25.30 | 6.55; 44 | 0.01* | 23.90 | 1.41; 46.3 | 0.04* | 26.00 | 8.54; 43.5 | 0.004* |
| Weight (pounds) | -0.51 | -2.01; 0.98 | 0.50 | 0.91 | -4.86; 6.67 | 0.76 | 0.35 | -6.43; 7.13 | 0.92 | 1.19 | -4.24; 6.61 | 0.67 |
| Height (meters) | 262.03 | -559.37; 1083.44 | 0.53 | -1268.00 | -4422; 1887 | 0.43 | -1480.00 | -5187; 2226 | 0.43 | -1161.00 | -4133; 1811 | 0.44 |

FA: Fractional Anisotropy; MD: Mean Diffusivity; AD: Axial Diffusivity; RD: Radial Diffusivity.

* Variable had a p-value of ≤ 0.20 and was a candidate for entry into the model; † Variable did not remain significant when entered in model.

**Appendix Table 4**: Results of the univariate linear regression for variables that could influence diffusion metrics (FA, MD, AD, and RD). Variables that were significant were candidates for use in the models including patients with TMD only, and all tracts except for the fornix

| | FA | | | MD | | | AD | | | RD | | |
|---|---|---|---|---|---|---|---|---|---|---|---|---|
| **Variable** | **Coef. (x10$^{-4}$)** | **95% CI (x10$^{-4}$)** | **p-value** | **Coef. (x10$^{-7}$)** | **95% CI (x10$^{-7}$)** | **p-value** | **Coef. (x10$^{-7}$)** | **95% CI (x10$^{-)7}$)** | **p-value** | **Coef. (x10$^{-7}$)** | **95% CI (x10$^{-7}$)** | **p-value** |
| Age (years) | -1.23 | -5.93; 3.47 | 0.61 | -9.45 | -13.50; -5.37 | <0.001* | -16.40 | -21.80; -10.90 | <0.001* | -6.01 | -11.20; -0.83 | 0.02* |
| Weight (pounds) | 0.50 | -0.92; 1.92 | 0.49 | 0.90 | -0.37; 2.17 | 0.16*† | 1.96 | 0.24; 3.67 | 0.03* | 0.37 | -1.20; 1.95 | 0.64 |
| Height (meters) | -208.31 | -1057.72; 641.09 | 0.63 | 584.00 | -173.00; 1342.00 | 0.13*† | 630.00 | -400.00; 1660.00 | 0.23 | 562.00 | -380.00; 1503.00 | 0.24 |
| Years of Complaint | 2.37 | -4.10; 8.84 | 0.47 | -9.54 | -15.20; -3.83 | 0.001*† | -11.70 | -19.50; -3.97 | 0.003*† | -8.44 | -15.60; -1.30 | 0.02*† |
| Chief Complaint (0=myogenous, 1=mixed) | -37.73 | -136.09; 60.62 | 0.45 | 59.50 | -28.40; 147.00 | 0.18*† | 56.80 | -62.60; 176.00 | 0.35 | 60.80 | -48.40; 170.00 | 0.28 |
| Other Complaints (0=no, 1=yes) | 47.00 | -52.45; 146.44 | 0.35 | -18.80 | -108.00; 70.30 | 0.68 | 26.40 | -94.50; 147.00 | 0.67 | -41.40 | -152.00; 69.10 | 0.46 |
| Other complaints (sum) | 8.26 | -45.79; 62.32 | 0.77 | -15.00 | -63.30; 33.30 | 0.54 | -16.10 | -81.80; 49.50 | 0.63 | -14.40 | -74.50; 45.60 | 0.64 |
| Other complaints – Neck (0=no, 1=yes) | 21.63 | -79.49; 122.74 | 0.68 | 64.50 | -25.70; 155.00 | 0.16*† | 116.00 | -5.83; 239.00 | 0.06*† | 38.60 | -73.70; 151.00 | 0.50 |

| | | | | | | | | | | | | |
|---|---|---|---|---|---|---|---|---|---|---|---|---|
| Other complaints – Headache (0=no, 1=yes) | 36.06 | -90.02; 162.14 | 0.58 | -101.00 | -214.00; 10.90 | 0.08* | -113.00 | -266.00; 40.00 | 0.15*† | -95.70 | -235.00; 44.00 | 0.18* |
| Other complaints – Shoulder (0=no, 1=yes) | -39.81 | -243.66; 164.05 | 0.70 | 98.50 | -83.60; 281.00 | 0.30 | 106.00 | -142.00; 353.00 | 0.40 | 95.00 | -131.00; 321.00 | 0.41 |
| Other complaints – Back (0=no, 1=yes) | -129.26 | -412.74; 154.22 | 0.37 | -168.00 | -422.00; 85.00 | 0.19*† | -431.00 | -773.00; -89.60 | 0.01*† | -36.70 | -352.00; 278.00 | 0.82 |
| Other complaints – Whiplash (0=no, 1=yes) | 12.04 | -97.20; 121.27 | 0.83 | -63.80 | -161.00; 33.70 | 0.20*† | -83.40 | -216.00; 49.00 | 0.22 | -54.00 | -175.00; 67.20 | 0.38 |
| Antidepressant (0=no, 1=yes) | 23.36 | -79.81; 126.54 | 0.66 | 83.10 | -8.77; 175.00 | 0.08*† | 152.00 | 27.80; 276.00 | 0.02* | 48.60 | -65.90; 163.00 | 0.41 |
| Active mouth open (mm) | -1.92 | -8.97; 5.14 | 0.59 | 3.45 | -2.86; 9.76 | 0.28 | 2.91 | -5.66; 11.50 | 0.51 | 3.72 | -4.11; 11.60 | 0.35 |
| Passive mouth open (mm) | -3.52 | -12.69; 5.64 | 0.45 | 4.77 | -3.42; 13.00 | 0.25 | 3.32 | -7.82; 14.50 | 0.56 | 5.50 | -4.67; 15.70 | 0.29 |
| VAS average | -0.82 | -3.32; 1.68 | 0.52 | -0.51 | -2.74; 1.73 | 0.66 | -1.39 | -4.42; 1.64 | 0.37 | -0.06 | -2.84; 2.71 | 0.96 |
| VAS current | -0.53 | -2.9; 1.83 | 0.66 | -0.47 | -2.59; 1.64 | 0.66 | -0.99 | -3.86; 1.88 | 0.50 | -0.22 | -2.84; 2.41 | 0.87 |
| VAS past week | -0.93 | -3.28; 1.42 | 0.44 | -0.43 | -2.53; 1.68 | 0.69 | -1.49 | -4.34; 1.36 | 0.31 | 0.10 | -2.51; 2.71 | 0.94 |
| PHQ-9 total score | -1.79 | -11.23; 7.65 | 0.71 | -4.43 | -12.90; 4.00 | 0.30 | -8.03 | -19.50; 3.41 | 0.17*† | -2.63 | -13.10; 7.85 | 0.62 |
| TOBCL total score | -1.99 | -8.62; 4.65 | 0.56 | -2.02 | -7.95; 3.92 | 0.51 | -4.95 | -13.00; 3.10 | 0.23 | -0.56 | -7.93; 6.82 | 0.88 |
| CSI total score | -1.73 | -7.15; 3.69 | 0.53 | 1.49 | -3.39; 6.37 | 0.55 | 0.77 | -5.78; 7.32 | 0.82 | 1.85 | -4.19; 7.90 | 0.55 |
| LODF total score | -0.83 | -8.09; 6.43 | 0.82 | -2.24 | -8.73; 4.25 | 0.50 | -3.90 | -12.70; 4.91 | 0.39 | -1.41 | -9.47; 6.65 | 0.73 |
| JFLS-20 total score | -0.63 | -2.79; 1.53 | 0.57 | -1.58 | -3.50; 0.35 | 0.11*† | -3.02 | -5.62; -0.41 | 0.02*† | -0.86 | -3.26; 1.53 | 0.48 |
| PHQ-15 total score | -0.65 | -12.42; 11.13 | 0.91 | -7.31 | -17.80; 3.20 | 0.17*† | -11.20 | -25.40; 3.09 | 0.12*† | -5.38 | -18.40; 7.69 | 0.42 |
| GCPS – Days in pain | 0.17 | -0.61; 0.95 | 0.66 | 0.03 | -0.67; 0.72 | 0.94 | 0.29 | -0.65; 1.23 | 0.55 | -0.10 | -0.97; 0.76 | 0.81 |
| GCPS – Disability interference days | 1.14 | -6.78; 9.07 | 0.78 | -5.43 | -12.50; 1.64 | 0.13*† | -6.41 | -16.00; 3.19 | 0.19*† | -4.93 | -13.70; 3.85 | 0.27 |

| | | | | | | | | | | | | |
|---|---|---|---|---|---|---|---|---|---|---|---|---|
| GCPS – Pain intensity | -0.33 | -2.71; 2.05 | 0.79 | -1.01 | -3.13; 1.12 | 0.35 | -1.60 | -4.49; 1.28 | 0.28 | -0.71 | -3.35; 1.93 | 0.60 |
| GCPS – Interference | -0.23 | -2.26; 1.80 | 0.82 | -1.83 | -3.63; -0.02 | 0.05*† | -2.96 | -5.40; -0.51 | 0.02*† | -1.26 | -3.51; 0.99 | 0.27 |
| GCPS – Disability points | -0.04 | -24.62; 24.53 | 1.00 | -23.20 | -45.00; -1.31 | 0.04*† | -34.70 | -64.40; -5.12 | 0.02*† | -17.40 | -44.60; 9.87 | 0.21 |
| NDI – total score | -2.35 | -11.67; 6.97 | 0.62 | -6.30 | -14.60; 2.02 | 0.14*† | -12.00 | -23.20; -0.72 | 0.04*† | -3.46 | -13.80; 6.89 | 0.51 |
| PPT hand | 5.75 | -34.88; 46.37 | 0.78 | 21.60 | -14.60; 57.90 | 0.24 | 37.50 | -11.70; 86.70 | 0.14*† | 13.70 | -31.40; 58.80 | 0.55 |
| PPT temporalis anterior left | 23.94 | -53.64; 101.51 | 0.55 | 14.50 | -54.90; 83.90 | 0.68 | 47.10 | -47.00; 141.00 | 0.33 | -1.83 | -88.00; 84.40 | 0.97 |
| PPT temporalis anterior right | 24.11 | -44.92; 93.14 | 0.49 | -8.36 | -70.20; 53.40 | 0.79 | 13.00 | -70.90; 96.90 | 0.76 | -19.10 | -95.70; 57.60 | 0.63 |
| PPT temporalis medial left | 35.51 | -28.33; 99.35 | 0.28 | 21.90 | -35.30; 79.10 | 0.45 | 73.50 | -3.74; 151.00 | 0.06*† | -3.92 | -74.90; 67.10 | 0.91 |
| PPT temporalis medial right | 49.90 | -13.34; 113.14 | 0.12* | -14.40 | -71.20; 42.40 | 0.62 | 36.80 | -40.20; 114.00 | 0.35 | -40.00 | -110.00; 30.40 | 0.27 |
| PPT temporalis posterior left | 6.41 | -49.27; 62.08 | 0.82 | 22.90 | -26.90; 72.60 | 0.37 | 41.10 | -26.40; 109.00 | 0.23 | 13.70 | -48.10; 75.50 | 0.66 |
| PPT temporalis posterior right | 26.85 | -34.08; 87.77 | 0.39 | 9.91 | -44.70; 64.50 | 0.72 | 46.00 | -27.90; 120.00 | 0.22 | -8.15 | -75.90; 59.60 | 0.81 |
| PPT masseter superior anterior left | 39.01 | -44.31; 122.34 | 0.36 | 45.10 | -29.40; 120.00 | 0.24 | 114.00 | 13.60; 215.00 | 0.03*† | 10.60 | -82.00; 103.00 | 0.82 |
| PPT masseter superior anterior right | 5.15 | -95.50; 105.79 | 0.92 | 109.00 | 20.00; 199.00 | 0.02*† | 172.00 | 51.20; 293.00 | 0.01*† | 77.90 | -33.50; 189.00 | 0.17*† |
| PPT masseter superior inferior left | 12.72 | -67.29; 92.73 | 0.76 | 23.20 | -48.30; 94.80 | 0.52 | 46.80 | -50.30; 144.00 | 0.34 | 11.50 | -77.40; 100.00 | 0.80 |
| PPT masseter superior inferior right | 40.57 | -53.93; 135.07 | 0.40 | 61.20 | -23.20; 146.00 | 0.16*† | 137.00 | 22.60; 251.00 | 0.02*† | 23.60 | -81.50; 129.00 | 0.66 |
| PPT masseter deep left | 25.30 | -62.45; 113.06 | 0.57 | 23.70 | -54.80; 102.00 | 0.55 | 64.00 | -42.50; 170.00 | 0.24 | 3.53 | -94.00; 101.00 | 0.94 |
| PPT masseter deep right | 21.45 | -61.98; 104.89 | 0.61 | 28.60 | -46.00; 103.00 | 0.45 | 64.60 | -36.60; 166.00 | 0.21 | 10.60 | -82.10; 103.00 | 0.82 |

FA: Fractional Anisotropy; MD: Mean Diffusivity; AD: Axial Diffusivity; RD: Radial Diffusivity; VAS: Visual Analog Scale; TOBCL: Oral Behavior Checklist; PHQ-9: Patient Health Questionnaire-9; PHQ-15: Patient Health Questionnaire-15; NDI: Neck Disability Index; LODF: Limitations of Daily Functions; JFLS-20: Jaw Functional Limitations Scale-20; CSI: Central Sensitization Inventory; GCPS: ; GCPS: graded chronic pain scale; PPT: Pressure Pain Threshold. * Variable had a p-value of ≤ 0.20 and was a candidate for entry into the model; † Variable did not remain significant when entered in model.

**Appendix Table 5**: Results of the univariate linear regression for variables that could influence diffusion metrics (FA, MD, AD, and RD). Variables that were significant were candidates for use in the model including patients with TMD only, and only the fornix.

| | FA | | | MD | | | AD | | | RD | | |
|---|---|---|---|---|---|---|---|---|---|---|---|---|
| **Variable** | **Coef. (x10$^{-4}$)** | **95% CI (x10$^{-4}$)** | **p-value** | **Coef. (x10$^{-7}$)** | **95% CI (x10$^{-7}$)** | **p-value** | **Coef. (x10$^{-7}$)** | **95% CI (x10$^{-)7}$)** | **p-value** | **Coef. (x10$^{-7}$)** | **95% CI (x10$^{-7}$)** | **p-value** |
| Age (years) | -5.20 | -10.49; 0.08 | 0.05* | 13.90 | -9.54; 37.3 | 0.25 | 11.30 | -17.9; 40.5 | 0.45 | 15.20 | -5.94; 36.3 | 0.16* |
| Weight (pounds) | -1.87 | -3.42; 0.08 | 0.02*† | 8.95 | 2.4; 15.5 | 0.007*† | 9.96 | 1.73; 18.2 | 0.02*† | 8.45 | 2.53; 14.4 | 0.005*† |
| Height (meters) | -521.51 | -1512.82; -0.31 | 0.30 | 4549.00 | 516; 8582 | 0.03*† | 5785.00 | 835; 10736 | 0.002*† | 3931.00 | 233; 7629 | 0.04*† |
| Years of Complaint | 0.0049 | -7.67; 7.68 | 1.00 | 4.93 | -28; 37.8 | 0.77 | 5.97 | -34.6; 46.5 | 0.77 | 4.41 | -25.5; 34.4 | 0.77 |
| Chief Complaint (0=myogenous, 1=mixed) | -64.96 | -179.52; 49.6 | 0.27 | 253.00 | -240; 746 | 0.32 | 284.00 | -326; 894 | 0.36 | 237.00 | -211; 686 | 0.30 |
| Other Complaints (0=no, 1=yes) | -27.46 | -145.11; 90.19 | 0.65 | 378.00 | -112; 868 | 0.13*† | 505.00 | -95.6; 1105 | 0.10*† | 315.00 | -133; 763 | 0.17*† |
| Other complaints (sum) | -27.86 | -91.22; 35.51 | 0.39 | 182.00 | -85.8; 450 | 0.18*† | 220.00 | -111; 550 | 0.19*† | 763.00 | -80.8; 407 | 0.19*† |
| Other complaints – Neck (0=no, 1=yes) | -18.64 | -138.35; 101.07 | 0.76 | 224.00 | -284; 733 | 0.39 | 301.00 | -325; 927 | 0.35 | 186.00 | -278; 650 | 0.43 |
| Other complaints – Headache (0=no, 1=yes) | 11.55 | -137.92; 161.01 | 0.88 | 65.30 | -576; 707 | 0.84 | 110.00 | -680; 900 | 0.78 | 43.00 | -541; 627 | 0.89 |
| Other complaints – Shoulder (0=no, 1=yes) | -7.95 | -249.62; 233.72 | 0.95 | -556.00 | -1575; 464 | 0.29*† | -807.00 | -2056; 442 | 0.21 | -430.00 | -1362; 503 | 0.37 |
| Other complaints – Back (0=no, 1=yes) | -397.01 | -704.95; -89.08 | 0.01* | 1475.00 | 122; 2828 | 0.03*† | 1500.00 | -205; 3205 | 0.08*† | 1462.00 | 247; 2678 | 0.02*† |
| Other complaints – Whiplash (0=no, 1=yes) | -39.57 | -168.35; 89.21 | 0.55 | 373.00 | -167; 914 | 0.18*† | 473.00 | -193; 1139 | 0.16*† | 324.00 | -170; 817 | 0.20*† |
| Antidepressant (0=no, 1=yes) | -162.49 | -271.53; -53.44 | 0.004* | 899.00 | 473; 1325 | <0.001* | 1049.00 | 510; 1589 | <0.001* | 824.00 | 438; 1210 | <0.001* |

| | | | | | | | | | | | | |
|---|---|---|---|---|---|---|---|---|---|---|---|---|
| Active mouth open (mm) | -4.82 | -13.02; 3.39 | 0.25 | 29.30 | -5.19; 63.8 | 0.10*† | 34.40 | -8.35; 77.1 | 0.11*† | 26.80 | -4.61; 58.2 | 0.09*† |
| Passive mouth open (mm) | -4.19 | -14.97; 6.59 | 0.45 | 24.40 | -21.5; 70.3 | 0.30 | 28.10 | -28.7; 84.8 | 0.33 | 22.60 | -19.2; 64.4 | 0.29 |
| VAS average | 0.24 | -2.72; 3.2 | 0.88 | -5.96 | -18.5; 6.58 | 0.35 | -8.17 | -23.6; 7.25 | 0.30 | -4.86 | -16.3; 6.59 | 0.41 |
| VAS current | -0.02 | -2.82; 2.78 | 0.99 | -5.39 | -17.3; 6.5 | 0.37 | -7.64 | -22.2; 6.96 | 0.31 | -4.26 | -15.1; 6.59 | 0.44 |
| VAS past week | 0.44 | -2.35; 3.22 | 0.76 | -5.24 | -17.1; 6.59 | 0.39 | -6.92 | -21.5; 7.64 | 0.35 | -4.40 | -15.2; 6.39 | 0.42 |
| PHQ-9 total score | 1.46 | -9.72; 12.63 | 0.80 | -13.80 | -61.6; 34 | 0.57 | -18.80 | -77.6; 40.1 | 0.53 | -11.30 | -54.8; 32.2 | 0.61 |
| TOBCL total score | -2.59 | -10.41; 5.24 | 0.52 | -6.82 | -40.5; 26.9 | 0.69 | -13.90 | -55.3; 27.5 | 0.51 | -3.29 | -34; 27.4 | 0.83 |
| CSI total score | -0.39 | -6.81; 6.03 | 0.90 | 3.77 | -21.5; 29 | 0.77 | 4.82 | -25.2; 34.8 | 0.75 | 3.24 | -20.3; 26.8 | 0.79 |
| LODF total score | -0.43 | -9.03; 8.17 | 0.92 | -1.05 | -38; 35.9 | 0.96 | -1.54 | -47.1; 44 | 0.95 | -0.80 | -34.4; 32.8 | 0.96 |
| JFLS-20 total score | -0.74 | -3.28; 1.81 | 0.57 | 1.96 | -8.99; 12.9 | 0.73 | 2.09 | -11.4; 15.6 | 0.76 | 1.90 | -8.07; 11.9 | 0.71 |
| PHQ-15 total score | -3.66 | -17.56; 10.25 | 0.61 | 3.19 | -56.7; 63.1 | 0.92 | -1.32 | -75.2; 72.5 | 0.97 | 5.45 | -49; 59.9 | 0.84 |
| GCPS – Days in pain | -0.0009 | -0.94; 0.94 | 1.00 | -0.46 | -4.49; 3.58 | 0.82 | -0.63 | -5.61; 4.36 | 0.81 | -0.37 | -4.04; 3.3 | 0.84 |
| GCPS – Disability interference days | 0.73 | -8.67; 10.11 | 0.88 | -19.70 | -59.5; 20 | 0.33 | -28.50 | -77.3; 20.2 | 0.25 | -15.30 | -51.6; 21 | 0.41 |
| GCPS – Pain intensity | -1.53 | -4.3; 1.25 | 0.28 | -1.64 | -13.7; 10.5 | 0.79 | -5.12 | -19.9; 9.71 | 0.50 | 0.11 | -10.9; 11.1 | 0.99 |
| GCPS – Interference | -0.02 | -2.43; 2.38 | 0.99 | -5.22 | -15.4; 4.93 | 0.31 | -7.73 | -20.2; 4.71 | 0.22 | -3.97 | -13.3; 5.32 | 0.40 |
| GCPS – Disability points | -2.66 | -31.78; 26.45 | 0.86 | -78.50 | -201; 43.6 | 0.21 | -123.00 | -271; 25.2 | 0.10*† | -56.20 | -168; 56 | 0.33 |
| NDI – total score | 1.32 | -9.72; 12.36 | 0.81 | -16.50 | -63.6; 30.6 | 0.49 | -22.20 | -80.2; 35.7 | 0.45 | -13.60 | -56.5; 29.3 | 0.54 |
| PPT hand | 12.44 | -35.53; 60.41 | 0.61 | -44.60 | -251; 161 | 0.67 | -44.30 | -299; 210 | 0.73 | -44.80 | -232; 143 | 0.64 |
| PPT temporalis anterior left | -19.27 | -111.04; 72.49 | 0.68 | 231.00 | -156; 618 | 0.24 | 311.00 | -165; 786 | 0.20*† | 191.00 | -162; 544 | 0.29 |

| | | | | | | | | | | | | |
|---|---|---|---|---|---|---|---|---|---|---|---|---|
| PPT temporalis anterior right | - 19.72 | -101.32; 61.88 | 0.64 | 289.00 | -48.2; 626 | 0.09*† | 384.00 | -29.5; 797 | 0.07*† | 242.00 | -67.2; 551 | 0.13*† |
| PPT temporalis medial left | -5.03 | -80.81; 70.76 | 0.90 | 112.00 | -211; 435 | 0.50 | 154.00 | -244; 552 | 0.45 | 90.90 | -204; 385 | 0.55 |
| PPT temporalis medial right | 7.88 | -67.3; 83.05 | 0.84 | 154.00 | -165; 472 | 0.34 | 229.00 | -161; 619 | 0.25 | 116.00 | -175; 408 | 0.43 |
| PPT temporalis posterior left | - 31.36 | -96.48; 33.76 | 0.35 | 195.00 | -80; 470 | 0.16*† | 232.00 | -108; 572 | 0.18*† | 177.00 | -73.9; 427 | 0.17*† |
| PPT temporalis posterior right | 5.92 | -66.35; 78.19 | 0.87 | 76.10 | -233; 385 | 0.63 | 119.00 | -261; 500 | 0.54 | 54.50 | -227; 336 | 0.70 |
| PPT masseter superior anterior left | - 45.39 | -143.05; 52.28 | 0.36 | 243.00 | -174; 659 | 0.25 | 279.00 | -236; 793 | 0.29 | 225.00 | -154; 603 | 0.24 |
| PPT masseter superior anterior right | - 65.97 | -183.13; 51.18 | 0.27 | 409.00 | -83.7; 901 | 0.10*† | 485.00 | -125; 1094 | 0.12*† | 371.00 | -77.4; 819 | 0.10* |
| PPT masseter superior inferior left | - 47.14 | -140.62; 46.34 | 0.32 | 285.00 | -110; 681 | 0.16*† | 337.00 | -152; 825 | 0.18*† | 260.00 | -99.9; 620 | 0.16*† |
| PPT masseter superior inferior right | - 31.84 | -143.45; 79.77 | 0.58 | 408.00 | -52.5; 869 | 0.08* | 532.00 | -33.3; 1097 | 0.07* | 346.00 | -75.5; 768 | 0.11*† |
| PPT masseter deep left | - 60.94 | -162.91; 41.03 | 0.24 | 252.00 | -186; 690 | 0.26 | 269.00 | -274; 812 | 0.33 | 243.00 | -155; 641 | 0.23 |
| PPT masseter deep right | - 39.22 | -137.24; 58.8 | 0.43 | 253.00 | -162; 669 | 0.23 | 303.00 | -210; 816 | 0.25 | 229.00 | -150; 607 | 0.24 |

FA: Fractional Anisotropy; MD: Mean Diffusivity; AD: Axial Diffusivity; RD: Radial Diffusivity; VAS: Visual Analog Scale; TOBCL: Oral Behavior Checklist; PHQ-9: Patient Health Questionnaire-9; PHQ-15: Patient Health Questionnaire-15; NDI: Neck Disability Index; LODF: Limitations of Daily Functions; JFLS-20: Jaw Functional Limitations Scale-20; CSI: Central Sensitization Inventory; GCPS: ; GCPS: graded chronic pain scale; PPT: Pressure Pain Threshold.* Variable had a p-value of ≤ 0.20 and was a candidate for entry into the model; † Variable did not remain significant when entered in model.
