# Supplementary material for: White Matter Diffusion Properties in Chronic Temporomandibular Disorders: An Exploratory Analysis
Source: Front Pain Res (Lausanne). 2022 Jun 21;3:880831. doi: 10.3389/fpain.2022.880831 (PMC9254396; doi:10.3389/fpain.2022.880831)
Supplement: Supplementary file 2 [file Data_Sheet_2.pdf]

Data analysis of differences in DTI measures between groups (TMD vs. healthy) for all tracts except the fornix

To determine whether there were differences between groups (i.e., TMD and healthy subjects) in all DTI measures, all tracts that had distinct laterality (right and left) were included. The fornix was not included in this model since the tractography for this tract is unique as it is not separated into distinct left and right sides. Therefore, a separate model was done for Fornix alone.

To construct the models for each DTI measure and test the differences between groups, random effects (intercepts and slopes) were added to the models and tested for appropriateness using the Akaike Information Criterion (AIC) and the Bayesian Information Criteria (BIC) [1]. In addition, covariates of interest (i.e., demographic variables available for both groups) were entered as fixed effects and tested similarly. We began each analysis by performing univariate regression analyses to determine the association between all available variables and each DTI metric. Variables associated with the diffusion metrics at a p-value of less than 0.2 were candidates for inclusion in the model to predict that diffusion metric. Some have suggested this p-value as a conservative criterion to involve all potential variables that could be significant in a multivariable regression model. More traditional alpha levels can fail in identifying variables that could be important. Additionally, we were interested in modelling how DTI metrics differ between the different tracts, sides, and groups. We added interaction terms to the models to determine the interaction between tracts, sides, and groups.

We built the models using a forward analysis, meaning that we began with the most basic model, and each time we included a new variable in the model, we performed a comparison between the new model and the prior model using a likelihood ratio test to determine which model was a better fit and the two models were compared using the AIC and BIC as described

above. If a variable significantly improved the model, it was retained, and any remaining variables were tested using the same method. Once the final model was determined (including all variables that made a significant contribution to the model), the estimates from the variables for that model and their associations with each dependent variable (FA, MD, AD, or RD) were presented along with their 95% CIs.

After we determined the final models predicting the DTI metrics in both groups and all tracts except the fornix, we performed contrasts to determine whether diffusion metrics on each tract's right and left sides differed between groups.

Data analysis of differences in DTI measures between groups (TMD vs. healthy) for the fornix only

Separate models were built to determine differences between groups (i.e., TMD and healthy subjects) in all DTI measures obtained from the fornix only. As explained previously, the fornix is a structure spanning both left and right sides, and therefore it was analyzed separately.

To construct the models for each DTI measure and test the differences between groups, random and fixed effects were added to the models and tested using AIC and BIC, as described above. We tested the entry of random effects in these models, but they did not improve the models and were not included. Covariates of interest (i.e., demographic variables available for both groups) were then entered as fixed effects and tested similarly. Variables associated with the diffusion metrics at a p-value of ≤ 0.2 were candidates for inclusion in the model to predict each diffusion metric. Because we could not add tracts (since only the fornix was modelled) or sides (because the fornix is not split into left and right), we did not add any interaction terms to these models.

As with the previous models, once the final model was determined, the estimates from the variables for the final model and their associations with each dependent variable (FA, MD, AD, or RD) were presented along with their 95% CIs.

Relationship between clinical variables and DTI measures in subjects with TMD for all tracts except the fornix

To determine whether any of the clinical variables measured were associated with DTI measures in subjects with TMD, all tracts that had distinct laterality (right and left) were included. Again, this model omitted the fornix since it was not separated into left and right sides. Therefore, a separate model was constructed for the fornix alone to test the association between clinical variables and DTI metrics in subjects with TMD.

To construct the models for each DTI measure and determine their associations with clinical variables in subjects with TMD, as with the previous models, random effects (intercepts and slopes) were added to the models and tested for appropriateness using the AIC and BIC. In addition, covariates of interest (i.e., clinical variables and demographic variables) were entered as fixed effects and tested similarly. As before, variables associated with the diffusion metrics at a p-value of less than 0.2 were candidates for inclusion in the model to predict each diffusion metric (i.e., FA, MD, AD, or RD). Additionally, we were interested in modelling how DTI metrics differ between the different tracts and sides, so we added interaction terms to the models to determine that interaction.

Relationship between clinical variables and DTI measures in subjects with TMD for the fornix only

Separate models were built to determine whether any clinical variables measured were associated with DTI measures obtained from the fornix alone in subjects with TMD.

To construct the models for each DTI measure and test the differences between groups, random and fixed effects were added to the models and tested using AIC and BIC, as described above. As with the previous analyses including only the fornix, we tested the entry of random effects in these models, but they did not improve the models and were not included. Covariates of interest (i.e., clinical variables as well as demographic variables) were entered as fixed effects and tested similarly. Once again, variables associated with the diffusion metrics at a p-value of less than 0.2 were candidates for inclusion in the model.

As before, because we could not add tracts (since only the fornix was included here) or sides (because the fornix is not split into left and right), we did not add any interaction terms to these models. Finally, the estimates from the variables for the best model and their associations with each dependent variable (FA, MD, AD, or RD) were presented with their 95% CIs.

References

[1] Burnham KP, Anderson DR (2004) Multimodel inference: understanding AIC and BIC in model selection. Sociological methods & research 33:261-304.
